# Quantitative comparison of within-sample heterogeneity scores for DNA methylation data

**DOI:** 10.1093/nar/gkaa120

**Published:** 2020-02-27

**Authors:** Michael Scherer, Almut Nebel, Andre Franke, Jörn Walter, Thomas Lengauer, Christoph Bock, Fabian Müller, Markus List

**Affiliations:** 1 Computational Biology, Max Planck Institute for Informatics, Saarland Informatics Campus, 66123 Saarbrücken, Germany; 2 Graduate School of Computer Science, Saarland Informatics Campus, 66123 Saarbrücken, Germany; 3 Department of Genetics/Epigenetics, Saarland University, 66123 Saarbrücken, Germany; 4 Institute of Clinical Molecular Biology, Kiel University, 24105 Kiel, Germany; 5 CeMM Research Center for Molecular Medicine of the Austrian Academy of Sciences, 1090 Vienna, Austria; 6 Department of Laboratory Medicine, Medical University of Vienna, 1090 Vienna, Austria; 7 Department of Genetics, Stanford University School of Medicine, Stanford, CA 94305, USA; 8 Big Data in BioMedicine Group, Chair of Experimental Bioinformatics, TUM School of Life Sciences, Technical University of Munich, 85354 Freising, Germany

## Abstract

DNA methylation is an epigenetic mark with important regulatory roles in cellular identity and can be quantified at base resolution using bisulfite sequencing. Most studies are limited to the average DNA methylation levels of individual CpGs and thus neglect heterogeneity within the profiled cell populations. To assess this within-sample heterogeneity (WSH) several window-based scores that quantify variability in DNA methylation in sequencing reads have been proposed. We performed the first systematic comparison of four published WSH scores based on simulated and publicly available datasets. Moreover, we propose two new scores and provide guidelines for selecting appropriate scores to address cell-type heterogeneity, cellular contamination and allele-specific methylation. Most of the measures were sensitive in detecting DNA methylation heterogeneity in these scenarios, while we detected differences in susceptibility to technical bias. Using recently published DNA methylation profiles of Ewing sarcoma samples, we show that DNA methylation heterogeneity provides information complementary to the DNA methylation level. WSH scores are powerful tools for estimating variance in DNA methylation patterns and have the potential for detecting novel disease-associated genomic loci not captured by established statistics. We provide an R-package implementing the WSH scores for integration into analysis workflows.

## INTRODUCTION

**Figure 1.**
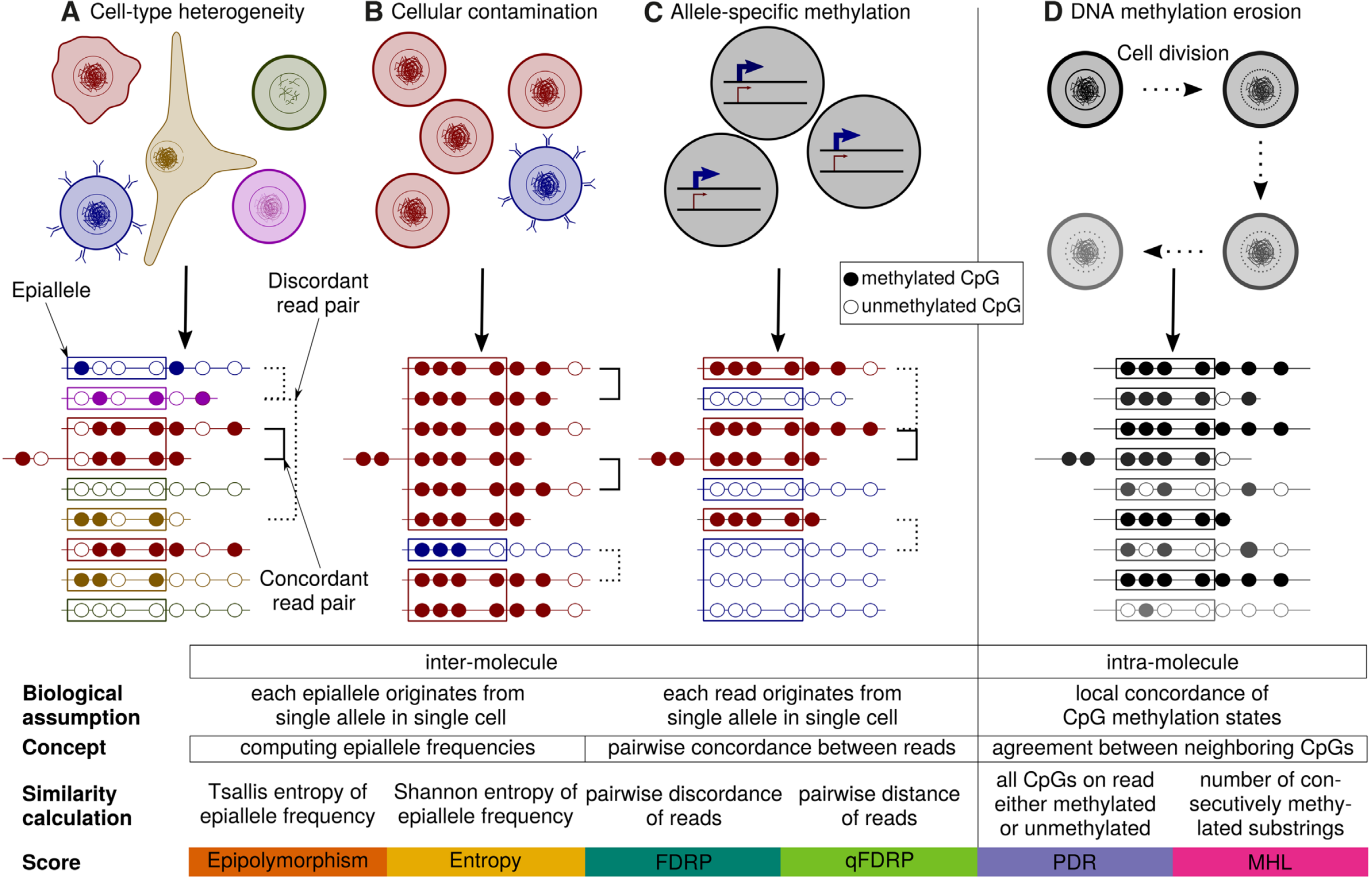
Sources of WSH and their manifestation in bisulfite sequencing reads. Each line represents a sequencing read. Characteristics of different WSH targeted at quantifying inter-molecule and intra-molecule heterogeneity are outlined below. (**A**) Cell-type heterogeneity: a sample comprises different cell types with different DNA methylation patterns. (**B**) Cellular contamination: sample preparation does not yield a pure population of cells of interest. (**C**) ASM: alleles differ in their DNA methylation states. (**D**) DNA methylation erosion: cells lose DNA methylation in a stochastic process. Epipolymorphism, Entropy, FDRP and qFDRP describe inter-molecule WSH (scenarios A–C), while PDR and MHL describe intra-molecule WSH (scenario D).

DNA methylation plays an important role in the regulation of gene expression, X-chromosome inactivation, genomic imprinting, and allele-specific expression (1–3), and it has been associated with a wide range of pathological states, such as cancer, developmental defects, premature aging diseases and healthy human aging (4–8). In mammalian cells, DNA methylation is predominantly found in CpG dinucleotides. Bisulfite sequencing captures DNA methylation states of individual cytosines (9,10) covering roughly $90\%$ of CpGs in whole-genome (WGBS) and approximately $10\hbox{--}20\%$ in reduced-representation bisulfite sequencing (RRBS). A single cytosine on a given DNA strand is either methylated or unmethylated and the DNA methylation level is quantified as the proportion of methylated molecules. However, about $2\%$ of 26.9 million CpGs in the human genome exhibit intermediate DNA methylation values at the level of bulk samples or tissues (11), which can be attributed to within-sample heterogeneity (WSH) (12). Potential sources of heterogeneity include cell-type composition (13,14), cellular contamination, technical issues, allele- and strand-specific DNA methylation (ASM and hemimethylation), and DNA methylation erosion, i.e. the stochastic loss of DNA methylation at a given locus (Figure 1).

While these heterogeneous methylation patterns can be deconvolved by physically separating cell types (5,15) or by *in-silico* approaches (16–18), we note that information about differences in DNA methylation heterogeneity between cell populations is valuable for understanding differences between phenotypes (12,19–20). Although single-cell bisulfite sequencing can be used to infer DNA methylation states of individual cells, high costs and technical issues such as low read numbers per cell currently prevent genome-wide heterogeneity analyses in larger studies. Alternatively, local deep amplicon sequencing can be used to reconstruct the probability distribution of DNA methylation patterns (21,22) but does not afford genome-wide coverage (23).

Various scores have thus been proposed for quantifying WSH from bulk bisulfite sequencing data: The *Proportion of Discordant Reads* (PDR) introduces the concept of locally disordered DNA methylation patterns (24) to quantify DNA methylation erosion (Figure 1D). Guo *et al.* (25) presented *Methylation Haplotype Load* (MHL) by defining methylation haplotypes as stretches of consecutively methylated CpGs. *Epipolymorphism* (26) and *Methylation Entropy* (27) (referred to as *Entropy* in the following) compute entropy in DNA methylation patterns of fixed size across sequencing reads. Notably, Epipolymorphism and Entropy take into account windows of multiple CpGs and therefore assume relative proximity of neighboring CpGs and neglect regions with low CpG density. To mitigate this, we propose the Fraction of Discordant Read Pairs (FDRP) and quantitative FDRP (qFDRP) which are the first scores for quantifying WSH at the level of individual CpGs. We particularly focus on scores that use raw bisulfite sequencing reads as input and do not discuss scores that quantify heterogeneity from processed DNA methylation data, such as the *proportion of sites with intermediate methylation* (PIM) or Shannon Entropy of particular markers (12,28–29).

Previous studies highlight the value of including WSH scores in standard DNA methylation analysis. For instance, PDR and Entropy were shown to be associated with gene expression (24) and transcriptional heterogeneity (30), as well as with important clinical parameters such as tumor size, progress-free survival and tumor location (12,31). However, choosing the most appropriate score for an analysis is not straightforward as a systematic review of WSH scores is currently missing in the literature. We therefore evaluated PDR, MHL, Epipolymorphism, Entropy, FDRP and qFDRP in the context of simulated and publicly available bisulfite sequencing data. We employed three criteria to evaluate performance: first, we tested if WSH scores cover different sources of heterogeneity (Figure 1). Second, we assessed robustness of WSH scores with respect to CpG density and technical biases such as sequencing coverage and read length. Third, we investigated the potential of WSH scores for revealing novel regulatory regions in particular those that are not apparent on the average DNA methylation level.

## MATERIALS AND METHODS

### Definitions of WSH scores

#### PDR

The PDR (24) models the local discordance between CpGs. It classifies each read as concordant if it shows consistent DNA methylation states at all CpG positions covered or as discordant otherwise. PDR is defined for a CpG *c* as:

$$\begin{eqnarray*} \displaystyle \text{PDR}(c) &=& \frac{\displaystyle \sum _{r\in R_c}I(\exists i,j\in r \text{ s.t. } x_{i,r}\ne x_{j,r})}{|R_c|}\\ r &=& \text{set of CpG positions (representing a read)}\\ R_c &=& \text{set of all reads }r\text{ covering } c\\ x_{i,r} \in \lbrace 0,1\rbrace &=& \text{methylation state of CpG } i \text{ in read } r \end{eqnarray*}$$

Note that the indicator function *I* is 1, if any two positions on the read (*i*, *j*) do not reflect the same DNA methylation state, i.e. the read is discordant and 0 otherwise. By definition reads are only included in the read set *R* if they contain at least four CpG sites.

#### MHL

The MHL (25) calculates the fraction of substrings of all possible lengths that are fully methylated in each of the reads. It is defined for a given CpG *c* as:

$$\begin{eqnarray*} \displaystyle \text{MHL}(c) &=& \frac{\displaystyle \sum _{l=0}^{L}(l+1){\frac{\displaystyle \sum _{r\in R_c}\displaystyle \sum _{i=1}^{ |r|-l}I(x_{i,r}=1 \wedge \cdots \wedge x_{i+l,r}=1)}{\displaystyle \sum _{r\in R_c}|r|-l}}}{\displaystyle \sum _{l=0}^{L}l+1}\\ x_{i,r} \in \lbrace 0,1\rbrace &=& \text{methylation state of CpG } i \text{ in read } r\\ R_c &=& \text{set of all reads }r\text{ covering } c\\ |r| &=& \text{number of CpGs in read } r\\ l &=& \text{number of consecutive CpGs of the same}\\ && \quad\text{methylation state}\\ L &=& \max _{r \in R_c}(|r|)-1 \end{eqnarray*}$$

#### Epipolymorphism and Methylation Entropy

Epipolymorphism (26) and Methylation Entropy (27) are based on *epialleles*, i.e. configurations of methylation states in four-CpG windows. The frequency of each of the 2^4^ possible epialleles is determined from the reads and Epipolymorphism for window $w$ is calculated as:

$$\begin{eqnarray*} \displaystyle \text{Epipolymor}\text{phism}(w) &=& 1 - \sum _{k=1}^{16} p_k^2\\ p_k &=& \frac{\displaystyle \sum _{r\in R_w}I(\forall i\in c_k: x_{i,c_k} = x_{i,r})}{|R_w|}\\ c_k &\in& \lbrace (0,0,0,0),(0,0,0,1),\ldots ,(1,1,1,1)\rbrace (\text{epiallele})\\ r &=& \text{set of CpG positions (representing a read)}\\ R_w &=& \text{set of all reads }r\text{ containing}\\ && \quad\text{all four CpGs in } w\\ x_{i,r} \in \lbrace 0,1\rbrace &=& \text{methylation state of CpG } i \text{ in read } r\\ x_{i,c_k} \in \lbrace 0,1\rbrace &=& \text{methylation state of CpG } i \text{ in epiallele } c_k\\ w &=& \text{window of four consecutive CpGs} \end{eqnarray*}$$

Using the above definitions, methylation entropy is calculated as:

$$\begin{eqnarray*} \displaystyle &&\text{Entropy}(w) = -\frac{1}{4}\sum _{k=1}^{16} p_k * \log _2{p_k} \end{eqnarray*}$$

#### FDRP and qFDRP

The FDRP captures within-sample DNA methylation heterogeneity at single CpG resolution. FDRP is defined as:

$$\begin{eqnarray*} \displaystyle \text{FDRP}(c)&=&\frac{\displaystyle \sum _{r_s\in R_c}\sum _{r_t \in R_c, t > s}I(\exists i\in \lbrace r_s \cap r_t\rbrace \text{ s.t. } x_{i,r_s} \ne x_{i,r_t})}{{|R_c|\atopwithdelims ()2}}\\ R_c &=& \text{set of all reads }r\text{ covering } c\\ r_s, r_t &=& \text{sets of CpG positions (representing reads)}\\ s, t \in [1,|R_c|] &=& \text{indices of reads} \\ x_{i,r} \in \lbrace 0,1\rbrace &=& \text{methylation state of CpG } i \text{ in read } r \end{eqnarray*}$$

FDRP is calculated from all read pairs that cover the sequence position of interest (*c*). We call a read pair discordant, if there is a CpG position in their overlap such that the methylation states differ. FDRP is normalized by the number of read pairs.

The quantitative FDRP (qFDRP) is derived from FDRP:

$$\begin{eqnarray*} \displaystyle \text{qFDRP}(c)&=& \frac{\displaystyle \sum _{r_s\in R_c}\sum _{r_t,t > s}\frac{\displaystyle \sum _{i \in \lbrace r_s\cap r_t\rbrace }I(x_{i,r_s} \ne x_{i,r_t})}{|\lbrace r_s\cap r_t\rbrace |}}{{|R_c|\atopwithdelims ()2}}\\ R_c &=& \text{set of all reads }r\text{ covering } c\\ r_s, r_t &=& \text{sets of CpG positions (representing reads)}\\ s, t \in [1,|R_c|] &=& \text{indices of reads} \\ x_{i,r} \in \lbrace 0,1\rbrace &=& \text{methylation state of CpG } i \text{ in read } r \end{eqnarray*}$$

In contrast to FDRP, qFDRP balances the discordance using the Hamming distance (Supplementary Data 1, Table S1). Note that the number of pairwise comparisons increases exponentially with coverage, necessitating a subsampling strategy (see ‘Implementation’ section).

### Implementation

FDRP and qFDRP were implemented in R (R-versions newer than release 3.2). We randomly sampled 40 reads at CpGs with coverage higher than 40 to avoid combinatorial explosion. We discarded read pairs with an overlap less than 35 bp, which removes reads with low information content. To account for different read lengths, we created a fixed-sized window (50 bp) around the CpG site of interest. We used RnBeads (32) data structures for storing DNA methylation, coverage and sample metadata. FDRP and qFDRP were calculated for sites having coverage at least 10 for the experimental data. In our freely available implementation, these parameters can be changed according to specific dataset characteristics.

PDR was computed with custom R scripts. Epipolymorphism and Methylation Entropy were calculated using *methclone* (version 0.1, (33)), which determines blocks of four adjacent CpGs and counts the epialleles found, using the following parameters: *methylation difference* 0, *distance cutoff* 50 bp and *coverage threshold* 10. The final scores were computed using custom R scripts. MHL was calculated by Perl scripts downloaded from the publication’s website (25).

The processing pipeline was implemented in python using *pypiper* (https://github.com/epigen/pypiper) and *looper* (https://github.com/epigen/looper) on a high-performance computing cluster. The pipeline scripts are available from GitHub (https://github.com/MPIIComputationalEpigenetics/WSHScripts). We provide an R package available from GitHub (https://github.com/MPIIComputationalEpigenetics/WSHPackage) implementing the scores discussed here together with an extensive vignette and manual on how to compute the scores from bisulfite sequencing data. FDRP/qFDRP calculation requires aligned reads in a bam file and genomic annotation through an RnBiseqSet or GRanges object (34) as input.

### Simulation of bisulfite sequencing reads and evaluation of WSH scores

#### Setup

We simulated bisulfite sequencing reads from the human reference genome ‘hg38’ (chromosome 22, X and Y excluded) using the Sherman tool (see Supplementary Data for details; https://www.bioinformatics.babraham.ac.uk/projects/sherman/). Sherman allows for controlling the methylation probability through the --CG_conversion parameter, which implicitly controls sample heterogeneity. We used different parameter settings for the different scenarios (Supplementary Data 1, Table S2).

Simulated reads were aligned to the reference genome ‘hg38’ with bismark (http://www.bioinformatics.babraham.ac.uk/projects/bismark/, version 0.13.0, (35)) to create bam files. DNA methylation scores were extracted and processed with RnBeads using default parameters. Bam files were sorted and indexed with samtools ((36), version 1.3).

#### Simulated heterogeneity

For each heterogeneity scenario (Figure 1), we simulated bisulfite sequencing reads in 1000 randomly selected genomic regions of length 50 kb using Sherman (read length: 50/100 bp; error level: $1\%$). For each region, we created different subpopulations of reads representing different cellular states which were subsequently merged to generate simulated samples for each region and scenario (Supplementary Data 1, Figure S1). For each subpopulation of reads, we assumed either a fully methylated or fully unmethylated (with error level $1\%$) background as baseline and introduced the opposite methylation state in a subset of CpGs within a randomly selected subregion. To assess the potential of each score to quantify heterogeneity, we performed a t-test comparing the different scores at each CpG in the background to the subregion. Additionally, we simulated negative cases in which no change in DNA methylation state for the subregion was introduced. In total we created 1000 simulated regions, which comprise a truly heterogeneous region (THR) in about half of the regions for each simulation scenario separately. We used these definitions to determine the numbers of true positives, true negatives, false positives and false negatives, as well as resulting Receiver Operating Characteristic (ROC) curves for each score and scenario (Supplementary Data 1, Figure S2).

In the cell-type heterogeneity scenario, we merged between 2 and 10 simulated cell types (read subpopulations). The subregions in which the methylation state changed to the inverse state were selected at random for each cell type individually. For each region, the THR was defined as the maximum segment at which any of the cell types changes from baseline DNA methylation level. Then, we computed the average WSH score for each of the 1000 regions individually and correlated this quantity with the number of cell types in this particular region to determine if the scores quantify the degree of heterogeneity in a sample. Cellular contamination was simulated using two distinct cell types, which were then mixed with a random proportion between 1.0 and 0.5. ASM was simulated analogously using 0.5 as a fixed proportion.

To simulate DNA methylation erosion, we first generated fully methylated regions for one cell type. For each region we randomly selected a subregion in which we demethylate CpGs with probability α. Since the eroded segments are passed on to all daughter cells in cell replication, we sampled from these demethylated reads between 2 and 10 times (parameter γ).

For methylation switching domains (MSDs), we modeled a single cell type with a baseline DNA methylation level. At a randomly selected position, the cell type changes its DNA methylation state.

#### Technical biases

To simulate coverage dependency, we randomly selected 1000 regions of size 50 kb and simulated different numbers of reads. Average DNA methylation levels were estimated from the blood cohort (see ‘Experimental Data’ section) with an overall average of $62.5\%$, where $42.4\%$ of all sites had methylation level of at least $95\%$ and $26.4\%$ had a DNA methylation value of at most $5\%$. We thus set the methylation probability to $62.5\%$ and the --CG_conversion parameter to 95 and 5 for the methylated and unmethylated states, respectively. For each of the regions, we selected between 5000 and 50 000 reads (step size 5000) for modeling coverage between roughly 5- and 50-fold. Then, we applied different read lengths (--length parameter) increasing from 40 to 150 bp (step size 10). To keep the simulated read coverage constant, we changed the number of reads generated according to the length parameter. We also employed different error levels (1–10%, step size $1\%$) to simulate sequencing errors. Note that the --error_rate parameter in Sherman employs an exponential decay model for each nucleotide with higher error probability for the 5’ than for the 3’ end (cf. Sherman manual at https://www.bioinformatics.babraham.ac.uk/projects/sherman/).

### Experimental data

RRBS data for 239 whole blood samples covering 5 606 227 CpGs at average read depth 7.5 from a healthy cohort (37) were obtained from the PopGen Biobank (https://www.uksh.de/p2n/). The dataset was used to validate simulation results and to estimate parameters for the simulation scenarios.

For further validation irrespective of the sequencing technology used, we collected WGBS data from the German Epigenome Program DEEP (http://www.deutsches-epigenom-programm.de), and we artificially created a homogeneous sample comprising hepatocyte populations from two distinct donors and a heterogeneous sample comprising mixed WGBS data from a hepatic cell line (HepaRG) and a primary hepatocyte sample. Primary data processing was performed according to the DEEP WGBS process documentation (https://github.molgen.mpg.de/DEEP/comp-metadata). Reads were trimmed using seqprep (https://github.com/jstjohn/SeqPrep), alignment was performed using methylCtools/bwa (38,39), and duplicates were removed using Picard (http://broadinstitute.github.io/picard/). The final dataset covered 23 290 153 sites at average read depth 24.1.

A Ewing sarcoma RRBS dataset (GSE88826) was used to illustrate the applicability of WSH scores in a disease context. This dataset comprised 188 samples with 140 Ewing tissue samples, 16 Ewing cell lines (Ewing CL), 21 mesenchymal stem cells extracted from healthy donors (MSCs) and 11 MSCs extracted from Ewing sarcoma patients (eMSCs) and covers 2 217 786 sites at average read depth 14.7. Both RRBS datasets were trimmed using TrimGalore (http://www.bioinformatics.babraham.ac.uk/projects/trim_galore) and aligned to reference genome version ‘hg38’ using bsmap (40).

### Quantification of WSH scores, tumor purity and differential heterogeneity

For all bisulfite datasets, we computed WSH scores as matrices of CpG-sites×samples. Scores were aggregated across samples or across annotated functional elements according to the Ensembl Regulatory Build (41). We discarded 11 formalin-fixed and paraffin-embedded (FFPE) samples from the Ewing tissue group, since they showed lower quality in the original publication (12). To predict tumor purity levels of the Ewing sarcoma dataset, we used the LASSO implemented in the glmnet R-package (42) to select sites linked to annotated purity levels for 81 of the 129 samples. Tumor purity levels were estimated from genetic data as described in (12). We used 10 random initializations of sample orderings and then employed 10-fold cross validation with different α-values ranging from 0 to 1. We subsequently repeated the process using sample permutations to further validate our findings. Predictive CpG sites were selected if they were on average present in five or more folds of the cross validation (Supplementary Data 1, Supplementary Text).

We transformed scores to *M*-values to call differentially methylated and differentially heterogeneous regions between two groups using limma (43) (both on single CpGs and after aggregation). After adjusting the resulting *P*-values for multiple testing with the Benjamini–Hochberg method (44), we set a FDR threshold of 0.01 and conducted enrichment analysis using GOstats (45) and LOLA (46). We report run time (wallclock time on a Debian 7 machine with 32 cores and 128 GB main memory) for each step (Supplementary Data 2).

## RESULTS

### Different WSH scores are designed to capture different biological sources of DNA methylation heterogeneity

The WSH scores discussed here are conceptually different (Figure 1; see ‘Materials and Methods’ section for mathematical definitions), and can be divided into two broad categories:

*Intra-molecule scores* like PDR and MHL are motivated by DNA methylation erosion and quantify the (dis)agreement between DNA methylation states of individual CpGs on the same read. PDR conceptualizes locally disordered DNA methylation patterns. A maximum score of 1 is obtained when all reads that cover a specific CpG contain both methylated and unmethylated CpGs. Conversely, PDR is 0 if all reads are consistently methylated/unmethylated. MHL defines DNA methylation haplotypes based on the CpG methylation states on a read. It is at its maximum (1) if all reads are fully methylated and at its minimum (0) if they are completely unmethylated. MHL does not increase linearly with the number of methylated CpGs but rather quantifies stretches of adjacently methylated CpGs.

*Inter-molecule scores* describe the variance in DNA methylation patterns at a given locus and are thus able to quantify cell-type heterogeneity. Epipolymorphism and Entropy utilize the concept of *epialleles* which describe patterns of DNA methylation states within four-CpG windows. They therefore assume spatial proximity of CpGs and they reach their maximum value (Epipolymorphism: $1-\frac{1}{16}=0.9375$, Entropy:$-\frac{1}{4}*16*\frac{1}{16}*\log _2(\frac{1}{16})=1$), if all 16 epialleles occur at the same frequency. Both scores are 0 if only a single pattern is represented. FDRP and qFDRP quantify the pairwise disagreement in methylation states of sequencing reads. They are at their maximum (1) if no two reads reflect the same DNA methylation pattern and are 0 if all reads show identical methylation patterns.

**Figure 2.**
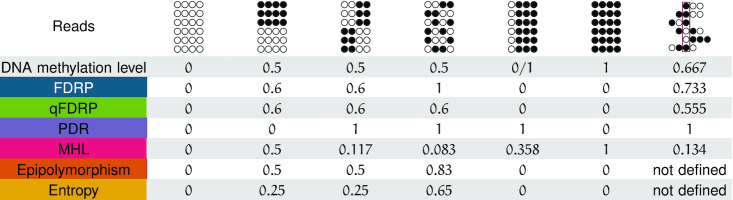
Comparison of average DNA methylation level and WSH scores for different sequencing read configurations.

We expect scores of the same class to show similar results (Figure 2). For example, Epipolymorphism and Entropy both compute epiallele frequencies and estimate epiallele variance using entropy. MHL, which directly quantifies stretches of consecutively methylated CpGs, is conceptually different from the other scores. In a local context, the human genome is mostly fully methylated or unmethylated. Hence, MHL and DNA methylation level describe similar characteristics.

Although defined as an intra-molecule score, PDR shares characteristics with qFDRP and FDRP (Figure 2; Supplementary Data 1, Table S1). All three scores rely on concordance between neighboring CpGs on the same read (PDR) or for the same CpG on different reads (FDRP/qFDRP). Nevertheless, the scores are designed to capture different biological phenomena: DNA methylation erosion (PDR) and cell-type heterogeneity (FDRP/qFDRP), respectively.

If a pair of reads contains many overlapping CpGs, the probability of detecting a single difference increases and results in a DNA methylation state mismatch in the FDRP definition. The qFDRP therefore computes the Hamming distance to quantify pairwise read differences and is thus less susceptible to DNA methylation fluctuations occurring at single CpGs.

### Inter-molecule WSH scores capture cell-type heterogeneity, cellular contamination and ASM in simulation experiments

#### Cell-type heterogeneity, cellular contamination and ASM

**Figure 3.**
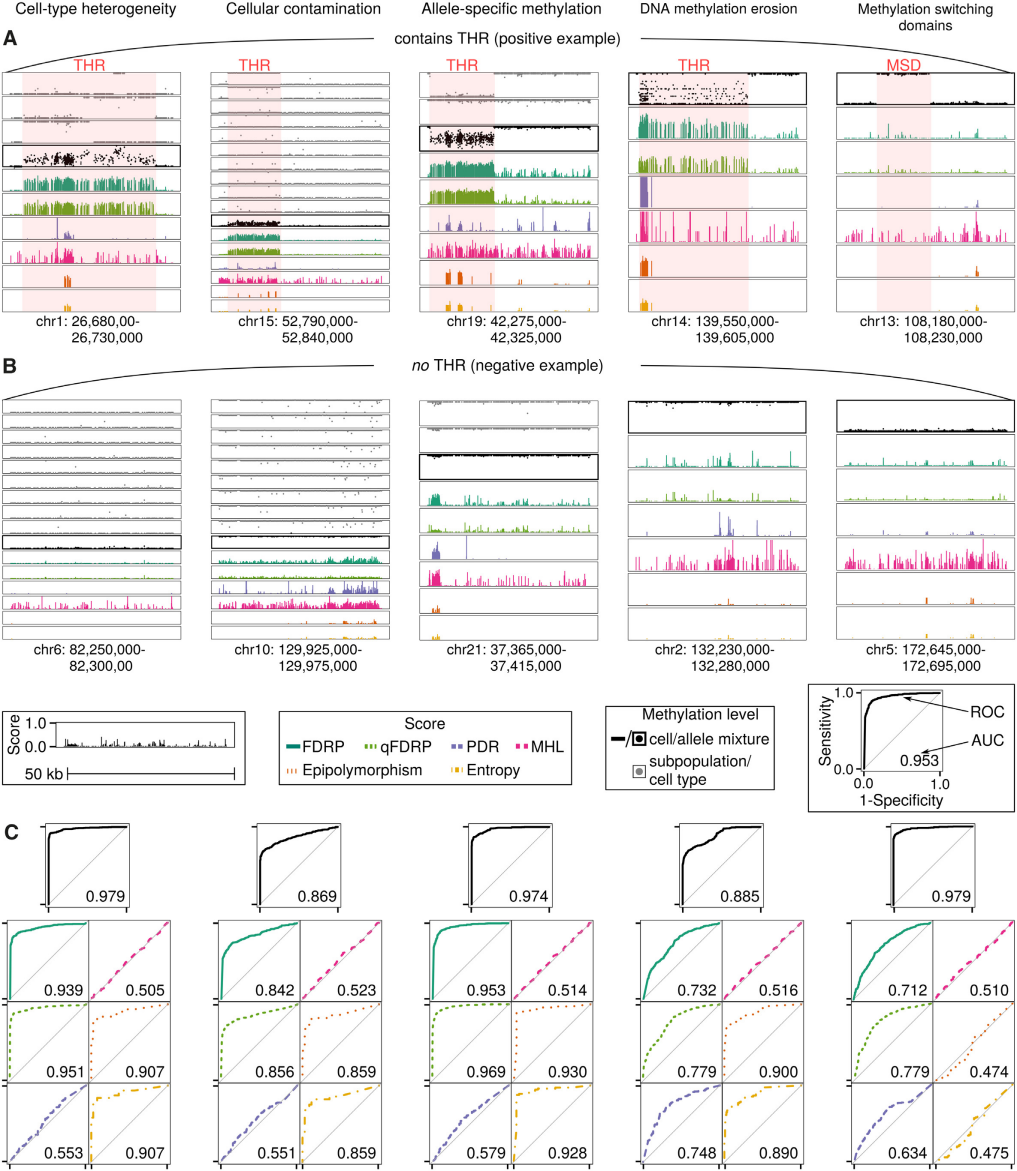
WSH scores in five simulation scenarios (50 bp reads): cell-type heterogeneity, cellular contamination, ASM, DNA methylation erosion and methylation switching domains. A positive (**A**) and negative (**B**) example is shown as snapshot for the scores and DNA methylation levels of single cell types (gray) and cellular mixture (black) for each scenario. 1000 regions of size 50 kb were simulated in total. (**C**) ROC curves represent whether the score/DNA methylation reliably differentiates THRs from the background using the *P*-value of a *t*-test.

Cell-type heterogeneity poses a challenge in bulk bisulfite sequencing of tissues. In our first scenario, we therefore merged 2–10 simulated cell types and evaluated the WSH scores’ ability to detect THRs. In the ‘cell-type heterogeneity’ scenario, we considered cell types at equal proportions while in the ‘cellular contamination’ scenario two cell types were mixed at different proportions. We found that the DNA methylation level, Epipolymorphism, Entropy, FDRP and qFDRP correctly identified the THRs in which the cell types exhibited distinct DNA methylation patterns (Figure 3), while the intra-molecule heterogeneity scores PDR and MHL were less accurate. Since Epipolymorphism and Entropy both require four CpGs per read, they were limited in their ability to quantify global WSH and could be quantified in only 70 out of the 1000 regions compared to FDRP/qFDRP which were quantifiable in 912 regions (Supplementary Data 1, Table S3). Furthermore, we found positive Spearman correlation coefficients of the average WSH scores per region and the number of simulated cell types for FDRP (0.61), qFDRP (0.61) and Epipolymorphism (0.28), but not for the average DNA methylation level (−0.03, Supplementary Data 1, Figure S3), suggesting that the WSH scores are better suited to capture the degree of heterogeneity compared to DNA methylation level alone.

Cell sorting methods do not always achieve a perfect separation, leading to the contamination of samples by non-target cell types. We therefore evaluated whether the WSH scores could detect such contamination in our simulated regions. qFDRP, FDRP, Epipolymorphism and Entropy quantified increased heterogeneity within the THR when we introduced between 0 and $50\%$ cell type contamination *in silico*. In contrast, MHL consistently exhibited high values in the background of the simulated regions, resulting in low AUC values. We observed higher scores for FDRP and PDR in CpG-dense regions (Figure 3). Similarly, the average DNA methylation level had high discriminatory power. Of note, due to their limitation to four contiguous CpGs, Epipolymorphism and Entropy were limited in their ability to quantify heterogeneity across all regions (Figure 3). MHL and PDR showed low accuracy in differentiating between THR and background. Correlating the mean WSH score with the simulated sample purity level per region, we found significant negative associations for FDRP (−0.47), qFDRP (−0.48), Epipolymorphism (−0.26) and Entropy (−0.47), but not for DNA methylation, PDR and MHL (Supplementary Data 1, Figure S3).

The diploid nature of mammalian genomes leads to additional complexity in DNA methylation patterns beyond cell-type heterogeneity. In particular, allele-specific methylation has been associated with allele-specific gene expression (3). In order to evaluate the scores’ capabilities to discern ASM from homogenous methylation, we simulated two artificial cell types (here representing alleles) at a 1 : 1 ratio similar to the two mixing scenarios above. With the exception of MHL and PDR, the WSH scores as well as the DNA methylation level accurately identified the THR in the majority of the 1000 regions. Notably, the above findings where consistent, when we changed the simulated read length from 50 to 100 bp (Supplementary Data 1, Figure S4).

#### DNA methylation erosion

We modeled erosion of DNA methylation originating from repeated cell divisions by introducing stochasticity in DNA methylation patterns in particular subregions. We found that the inter-molecule heterogeneity scores, which are designed to capture cell-type heterogeneity, also capture DNA methylation erosion in around two thirds of the simulated regions (Supplementary Data 1, Table S3). PDR, which was specifically designed for intra-molecule heterogeneity, performed more accurately than in the cell-mixture scenarios. When correlating the scores to the simulation parameters (Supplementary Data 1, Figure S1), we expected PDR, which is designed for detecting DNA methylation erosion, to be highest at α values close to 50 and for low γ values. Consequently, we found a positive correlation of α, the stochasticity parameter quantifying the degree of DNA methylation erosion, for PDR, but also for FDRP, qFDRP, Epipolymorphism and Entropy (Supplementary Data 1, Figure S3). However, we detected negative correlations between γ, the replication parameter specifying how often a particular pattern is found in the reads, and FDRP, qFDRP, Epipolymorphism and Entropy as expected, but not for PDR.

#### Methylation switching domains

WSH scores were designed to quantify complex DNA methylation patterns rather than simply identifying domains with distinct DNA methylation levels. Given that the average DNA methylation level could be used to accurately detect THRs in the above scenarios, we tested whether WSH scores specifically capture heterogeneity rather than switches in the DNA methylation level. We therefore assessed each score’s performance in detecting methylation switching domains (MSDs), i.e. regions that change the methylation state from fully methylated to unmethylated or vice versa, which is typically the case at boundaries of active regulatory elements. We simulated a single cell type and expected low WSH scores as MSDs do not represent DNA methylation heterogeneity. Consistent with this expectation, we observed a substantially inflated false-negative rate (Figure 3; Supplementary Data 1, Table S3) thus illustrating that WSH scores indeed contribute additional information to the DNA methylation level. We then checked whether the results obtained on these heterogeneity scenarios were confounded by differences in CpG-wise coverage or CpG density of the regions. Coverage was constant at 15× and only few regions showed very high or low coverage. The average number of CpGs per kb was 10, while few regions showed substantially higher density, probably representing CpG islands (Supplementary Data 1, Figure S5).

### Inter- and intra-molecule WSH scores share information

In order to quantify similarities between the different WSH scores, we merged all regions from the scenarios above and computed pairwise correlation coefficients (Supplementary Data 1, Figure S6). We observed relatively high correlation of qFDRP, FDRP, Epipolymorphism and Entropy with the intra-molecule WSH score PDR. This indicates shared information between locally disordered DNA methylation and inter-molecule differences. Correlation coefficients of 0.65–0.67 between FDRP/qFDRP and Epipolymorphism/Entropy indicate that the two groups of scores largely describe similar aspects of the DNA methylation landscape, while there are also distinct regions showing differences across the scores. MHL was unrelated to the other scores. With the exception of MHL and PDR, the WSH were generally higher in regions exhibiting overall intermediate methylation (Supplementary Data 1, Figure S6).

### qFDRP and MHL are robust with respect to technical biases

Next, we systematically simulated bisulfite sequencing reads from samples with differences in technical setup including read coverage, read length and sequencing errors, as well as CpG density. We disregarded differences in absolute values between the scores and focused on the scores’ relationship with respect to varying technical parameters.

**Figure 4.**
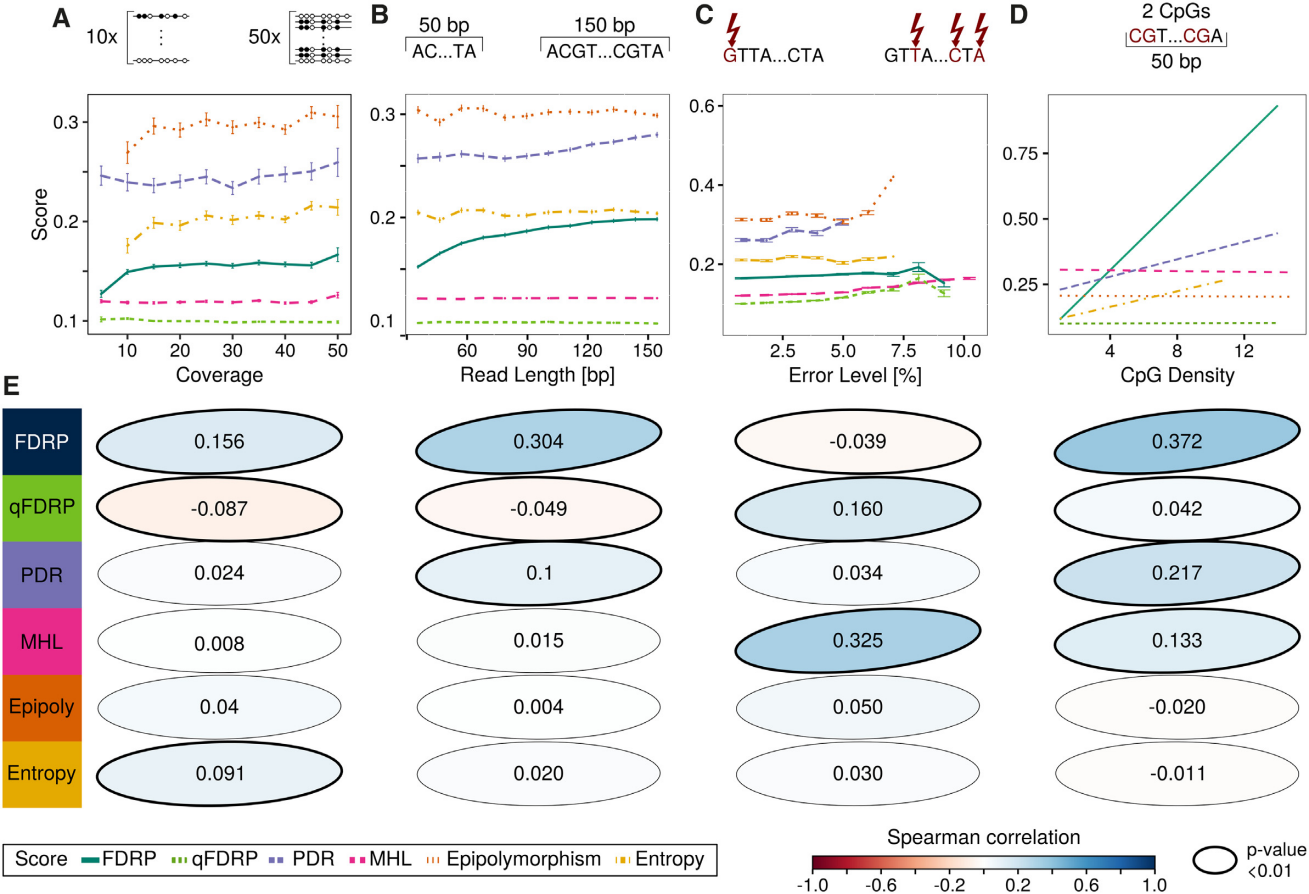
Effect of technical parameters on WSH scores in simulation experiments with 50 bp reads. Mean WSH scores are plotted against (**A**) the mean coverage across simulated regions, (**B**) the length of the simulated reads, (**C**) the simulated sequencing error level in percent and (**D**) the least squares regression line for the number of CpGs in a 50 bp window (CpG density). Error bars indicate standard error. (**E**) Spearman correlation coefficients to technical parameters are shown with ellipses that are directed toward the upper right for positive, and to the lower right for negative correlations. The color represents the magnitude of correlation and significant correlations (*P*-values < 0.01) are indicated by bold borders.

Coverage differences in samples present a major challenge for downstream analysis of bisulfite sequencing data (47). We therefore simulated bisulfite sequencing data at different read depths and determined the minimal coverage needed as well as potential coverage bias in WSH scores. Epipolymorphism and Entropy required coverage higher than 10-fold (Figure 4A). While FDRP, Epipolymorphism and Entropy increased with coverage, MHL, PDR and qFDRP were more consistent in their quantification across different coverage values. These results were consistent when we used 100 bp instead of 50 bp reads (Supplementary Data 1, Figure S7). Since coverage can vary considerably across the genome, we computed Spearman’s rank correlation of the coverage at individual CpGs and the WSH scores in an individual dataset (i.e. an individual region with a defined number of reads). None of the scores showed a dependency on CpG-wise coverage (Supplementary Data 1, Figure S8), indicating that all scores are applicable to comparing heterogeneity in regions with potentially different coverages, but some are not applicable to comparing datasets with different average coverage.

Since the length of sequencing reads can differ between bisulfite sequencing datasets, we investigated dependencies of the scores on read length. When we simulated reads with lengths between 40 and 150 bp, PDR and FDRP increased with longer reads while qFDRP and MHL appeared more independent of read length (Figure 4B). Epipolymorphism and Entropy had no obvious relation to read length but their values fluctuated for shorter reads.

Similarly, we investigated sensitivity of the scores to sequencing errors. FDRP and qFDRP were consistent in their quantifications up to an error level of $7 \%$, when they started to show inflated scores due to sequencing errors (Figure 4C). Epipolymorphism, Entropy and PDR exhibited this behavior at a lower error level. qFDRP, MHL, Epipolymorphism and Entropy were stable up to $5 \%$ error level before different DNA methylation states caused by sequencing errors were considered as heterogeneity.

For modeling potential dependencies of WSH scores on genomic base composition, we correlated the local CpG density as the number of CpGs in 50 bp sliding windows with the average WSH score. FDRP, PDR and MHL were correlated with local CpG density (Figure 4B; Supplementary Data 1, Figures S7 and 8), indicating a dependency on the number of CpGs that could potentially bias these scores in certain sequence contexts. qFDRP, which was designed to mitigate this issue, as well as Epipolymorphism and Entropy did not depend on local CpG density in our simulation experiments.

### WSH scores computed for bisulfite sequencing datasets corroborate simulation experiments

We validated the findings of our simulations on a human whole blood cohort of healthy individuals assayed with RRBS (37). First, we found that most of the computing time was used for MHL’s computation (Supplementary Data 2). Correlations detected among the scores, especially between intra- and inter-molecule scores, were even higher than those on simulated data, further emphasizing that locally disordered methylation and variety in DNA methylation patterns coincide (Supplementary Data 1, Figure S9). While Epipolymorphism and Entropy were highly correlated to qFDRP and FDRP, we found that qFDRP and FDRP captured more than twice as many regions (Supplementary Data 1, Table S4). Next, we constructed a homogeneous and heterogeneous sample *in-silico* by mixing similar (hepatocytes) or distant (hepatocytes and liver cell lines) cell types using WGBS data. Our results showed that all scores, except for MHL, exhibit elevated heterogeneity in the heterogeneous sample (Supplementary Data 1, Figures S10 and 11).

Unexpectedly, Epipolymorphism showed a negative association with coverage and MHL showed a strictly bimodal distribution (Supplementary Data 1, Figure S12). Heterogeneity was preferentially located in distal, rather than proximal regulatory elements defined by the Ensembl Regulatory Build (Supplementary Data 1, Figures S10 and 12).

### qFDRP identifies differentially heterogeneous regions in cancer

**Figure 5.**
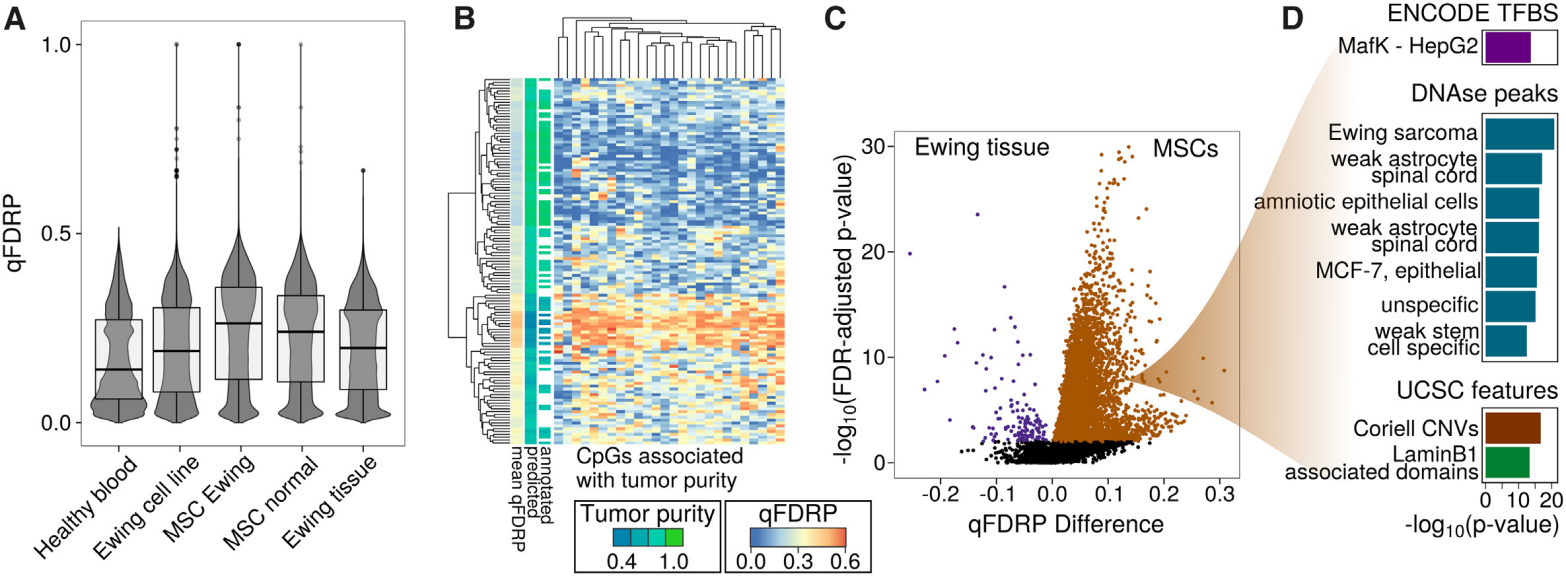
WSH in Ewing sarcoma samples. (**A**) Box- and violin plot of qFDRP values for different sample groups. (**B**) Heatmap of CpG-wise qFDRP scores (Ewing tissue samples without FFPE samples) for the 26 sites associated with tumor purity. Both samples (rows) and CpGs (columns) were hierarchically clustered according to Euclidean distance and complete linkage. The color code for the samples indicates predicted and annotated tumor purity levels, as well as the average qFDRP value across the selected sites. (**C**) Volcano plot of qFDRP values aggregated over genes in Ewing tissue samples versus MSCs. Positive values on the x-axis indicate higher WSH in MSCs. Each point is a gene, which is marked brown/purple if it has FDR-adjusted *P*-value not more than 0.01. (**D**) LOLA (46) enrichment analysis of MSC-hyper-heterogeneous genes. Histograms indicate negative logarithm of the enrichment p-value. TFBS: transcription factor binding sites, MCF-7: breast cancer cell line, FDR: false discovery rate, CNV: copy number variation

To investigate to what extent WSH scores are informative in a disease context, we quantified DNA methylation heterogeneity in a dataset comprising Ewing sarcoma samples (Figure 5; Supplementary Data 1, Figures S13–19). We stratified samples into Ewing tissue, Ewing cell lines (CL), and normal mesenchymal stem cells (MSC) as the potential cell-of-origin population for Ewing tumors (48). We distinguish between MSCs, which were obtained from healthy donors, and eMSCs, which originate from Ewing sarcoma patients (12). When we compared Ewing sarcoma to the healthy samples from the blood cohort, qFDRP indicated higher overall heterogeneity in the set of cancer samples (Figure 5A). In particular, we detected highest heterogeneity in MSCs and slightly higher values in eMSCs (mean: 0.229) compared to normal MSCs (mean: 0.214). Lower qFDRP values were found in the Ewing CLs (mean: 0.185) and Ewing tissue samples (mean: 0.184). MSCs were consistently the most heterogeneous samples across all the scores (except for MHL), and lowest heterogeneity was detected in healthy blood samples using PDR, FDRP and qFDRP (Supplementary Data 1, Table S5). Furthermore, lowest WSH was detected in TSS for all scores, including the average DNA methylation level (Supplementary Data 1, Figures S13–19).

Estimates of tumor purity are crucial for downstream analysis in cancer research but are not always annotated. This motivated us to test whether tumor purity can be predicted reliably using WSH scores. We trained a LASSO model that selected 26 sites significantly associated with tumor purity (Supplementary Data 1, Table S6). Using qFDRP, we could demonstrate good prediction performance with an overall cross-validated (CV) mean absolute error of 0.027 at a correlation of 0.966. This result was substantially better than the results obtained using other scores and the DNA methylation level (Supplementary Data 1, Table S7). Subsequently, we used this model to predict tumor purity levels of the 48 samples lacking annotation and found that they clustered well with the remaining samples (Figure 5). Overall, we found that the clustering was mainly driven by the tumor purity levels as expected, and that the qFDRP values of the selected sites were significantly higher in the low purity cluster than in the high purity cluster (mean 0.133 versus 0.291, *t*-test *P*-value < 2.2*10^−16^). When repeating this analysis with random sample label permutations, results were poor (CV correlation: 0.02, CV mean absolute difference: 0.13), demonstrating that our model in fact captured tumor purity.

In order to identify differential WSH between MSCs and Ewing tissue samples, we conducted differential analyses using hierarchical linear models (43). MSCs (both normal MSCs and eMSCs) exhibited globally higher heterogeneity than tissue samples. Genes that had higher average qFDRP in MSCs compared to Ewing tissue (hyper-heterogeneous in MSCs; Figure 5C, Supplementary Data 3) enriched for DNase-hypersensitive regions in various cell types. Intriguingly, we detected enrichment for DNase-hypersensitive sites linked to Ewing sarcoma for qFDRP and the DNA methylation level (cf. Figure 5D). We found different enrichments of qFDRP and methylation level in transcription factor binding sites (TFBS): while qFDRP enriched for *MafK*, differential DNA methylation levels were detected in binding sites of *c-MYC*, *c-FOS* and *GATA3* (Supplementary Data 1, Figure S19), which further emphasized that qFDRP captures a different aspect of the DNA methylome.

## DISCUSSION

We evaluated the performance of six scores for capturing within-sample heterogeneity in both simulation experiments and bisulfite sequencing datasets. In the simulations, the power of scores to detect heterogeneity varied depending on their design motivation. For instance, PDR did not capture inter-molecule heterogeneity, since it was created to capture intra-molecule heterogeneity. Intriguingly, PDR did not perform better than inter-molecule heterogeneity scores in detecting DNA methylation erosion in our simulation experiments. MHL identifies DNA methylation haplotype blocks in bisulfite sequencing reads and might thus not be suitable for capturing WSH according to our definition. PDR, Epipolymorphism and Entropy require four CpGs per sequencing read, which potentially masks regions of low CpG content. Other scores such as the qFDRP do not possess this limitation by definition and are thus more robust to technical variation.

Since read lengths shorter than 50 bp are rarely used, qFDRP, MHL, Epipolymorphism and Entropy can be applied to datasets of any read length and also for comparison of datasets with different read lengths. All scores were sensitive to sequencing errors in our simulations, but tolerated error levels up to $5 \%$, which is below error percentages reported for Illumina sequencing ($0.5\hbox{--}2 \%$, (49)). We note that the influence of experimental biases such as different laboratories, restriction enzymes, differences in the genomic coverage of WGBS and RRBS, or PCR duplication artifacts were not part of our simulations and remain to be investigated.

To validate simulation results and to show potential applications in a clinical setting, we analyzed three experimental datasets. We showed that tools developed for identifying differential DNA methylation levels between groups of samples, such as hierarchical linear models, can also be applied for quantifying differential heterogeneity. Using these methods, we found that the mesenchymal stem cells used here showed higher WSH than tumor samples. This can be explained by stem cells forming heterogeneous populations of cells, whereas tumor cells follow a more clonal behavior, or by technical issues in sample preparation. Another explanation are DNA methylation oscillations in primed ESCs, which are caused by increased expression of DNMT3A/B together with high expression of TET enzymes (50). We showed that qFDRP can be used to accurately predict tumor purity levels estimated from genetic data, which can be valuable if such data is missing. Moreover, qFDRP was the the best score to reliably predict tumor purity levels. As expected, higher heterogeneity was detected in those samples that had lower tumor purity estimates.

We found elevated WSH scores in regions not yet annotated to a functional category in the Ensembl regulatory build in all three bisulfite sequencing datasets as reported in (51). These regions could not be detected using the average DNA methylation level and their functional role and connection to diseases warrant further investigation. We envision that WSH scores can be used to segment the genome into regions with particularly high or low heterogeneity which can be characterized subsequently. PDR could also be employed to quantify reduced correlation of neighboring CpGs in cancer (52). To investigate to what extent cell-type heterogeneity influences WSH, the scores proposed here could be used in conjunction with DNA methylation based cell-type deconvolution tools (17,18).

### Recommendations and guidelines

Table 1 summarizes strengths and limitations of WSH scores in capturing different characteristics of heterogeneity. PDR is well suited for detecting locally disordered regions in large cancer datasets. However, one should be aware of its dependency on data quality and local base composition, especially read length and CpG density. Additionally, its dependency on four CpGs in a window limits its applicability to shorter read lengths. PDR and FDRP showed sensitivity to technical setup because of the strict classification of each read (pair) as discordant/concordant. qFDRP is particularly suitable for identifying regions exhibiting high heterogeneity due to cell-type differences, and complements CpG-level DNA methylation measurements. It also proved to be robust with respect to technical noise in our simulation setup. Epipolymorphism and Entropy are suitable for region-based analysis while they fail to capture heterogeneity in CpG-sparse regions. Like PDR, they are restricted to regions with at least four CpGs per read. MHL was less specific in quantifying WSH. While it was robust to technical variation in synthetic data, it did not correlate to the DNA methylation level and was only weakly correlated to the other scores.

**Table 1.** General guidelines for WSH scores

Score	Concept	Strengths	Drawbacks	Application scenario
**PDR**	Locally disordered methylation	Detects DNAm erosion; CpG-wise score; Fast computation	Simulated heterogeneity *not* detected; Dependency on read length and CpG density	Addressing locally disordered DNA methylation in large cancer datasets
**MHL**	Methylation haplotypes	CpG-wise score; Robust to technical setup	Simulated heterogeneity *not* detected; Slow computation	Linking genetically detected haplotypes to DNA methylation haplotypes
**Epipoly**	Variance among the reads	Simulated heterogeneity detected; Robust to technical setup	*no* CpG-wise score; Few regions captured	Segmentation into highly and lowly variably methylated regions for large bisulfite sequencing datasets
**Entropy**	Variance among the reads	Simulated heterogeneity detected; Robust to technical setup	*no* CpG-wise score; Few regions captured	Segmentation into highly and lowly variably methylated regions for large bisulfite sequencing datasets
**FDRP**	Variance among the reads	Simulated heterogeneity detected CpG-wise score	Dependency on coverage, read length and CpG density; Rather slow computation	Linking CpG-wise methylation values to epigenetic heterogeneity in large bisulfite sequencing datasets
**qFDRP**	Variance among the reads	Simulated heterogeneity detected; Robust to technical setup; CpG-wise score	Rather slow computation	Linking CpG-wise methylation values to epigenetic heterogeneity in large bisulfite sequencing datasets

## CONCLUSION

WSH scores provide insights into sample composition and cell subpopulations. Thus, they complement the DNA methylation level by revealing differences among individual cells and alleles with unknown functional impact. Nevertheless, to date WSH is rarely considered in epigenomic studies. Here, we provide the first systematic and comprehensive evaluation of WSH scores that capture DNA methylation pattern variations directly from the sequenced reads. Based on simulations and experimental data, we provide guidelines for selecting the WSH score most appropriate for complementing DNA methylation levels as surrogate of heterogeneity. Our results indicate that WSH scores are suitable for the identification of genomic regions in which DNA methylation heterogeneity drives phenotypic changes in development and disease.

## DATA AVAILABILITY

The Ewing dataset analyzed in the current study is available in the GEO repository, accession number GSE88826. DEEP data is available through EGA, accession number EGAD00001001865. The blood cohort samples were obtained from the PopGen Biobank (Schleswig-Holstein, Germany). They can be accessed through a Material Data Access Form and the application is available at http://www.uksh.de/p2n/Information+for+Researchers.html. The code used here is available from GitHub (https://github.com/MPIIComputationalEpigenetics/WSHScripts).

## Supplementary Material

gkaa120_Supplemental_Files
