# Supplementary material for: Quantitative comparison of within-sample heterogeneity scores for DNA methylation data
Source: Nucleic Acids Res. 2020 Feb 27;48(8):e46. doi: 10.1093/nar/gkaa120 (PMC7192612; doi:10.1093/nar/gkaa120)
Supplement: gkaa120_Supplemental_Files [file gkaa120_supplemental_files.zip › supplement.pdf]

Supplementary Data To
Quantitative Comparison of Within-Sample Heterogeneity Scores for DNA Methylation Data

Michael Scherer, Almut Nebel, Andre Franke, Jörn Walter, Thomas Lengauer, Christoph Bock, Fabian Müller[†], and Markus List[†]

[†] contributed equally

Supplementary Figures

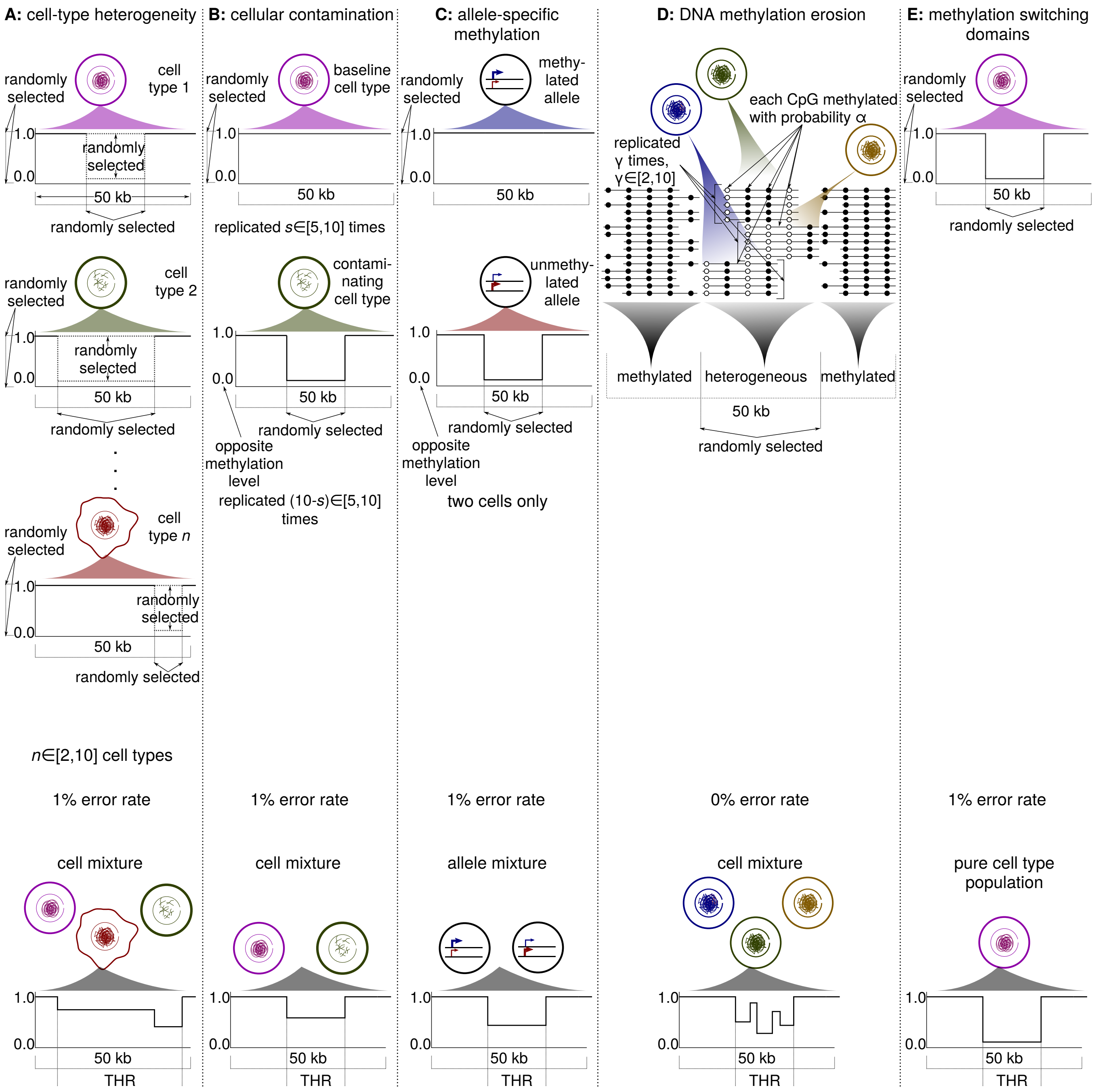

Figure S1: Simulation setup for the heterogeneity scenarios. Each region of size 50 kb contains multiple subpopulations/cell types (each row), and the truly heterogeneous region (THR) within each region is defined by the states of the cell types. The shaded areas visualize the dataset generated for each of the artificial cell types. **A** Between 2 and 10 simulated cell types (number of cell types n) were mixed for each region, each with a randomly selected DNA methylation state within a randomly selected sub-region. The THR is defined as the union of all of these sub-regions. **B** A pure cell population is mixed with 10-50 % of a contaminating cell type (sample purity level $s \in [0.5, 1]$). **C** Two cell types were generated, with one changing its DNA methylation state in a random sub-region. **D** In a randomly selected sub-region, all CpGs on a given read are de-methylated with the same probability (α). Those reads are then replicated γ times to represent the stochasticity of selecting cells for sequencing in a population of eroding cells. **E:** No heterogeneity was introduced. In a single cell type, the DNA methylation state changes from fully methylated to unmethylated or vice versa.

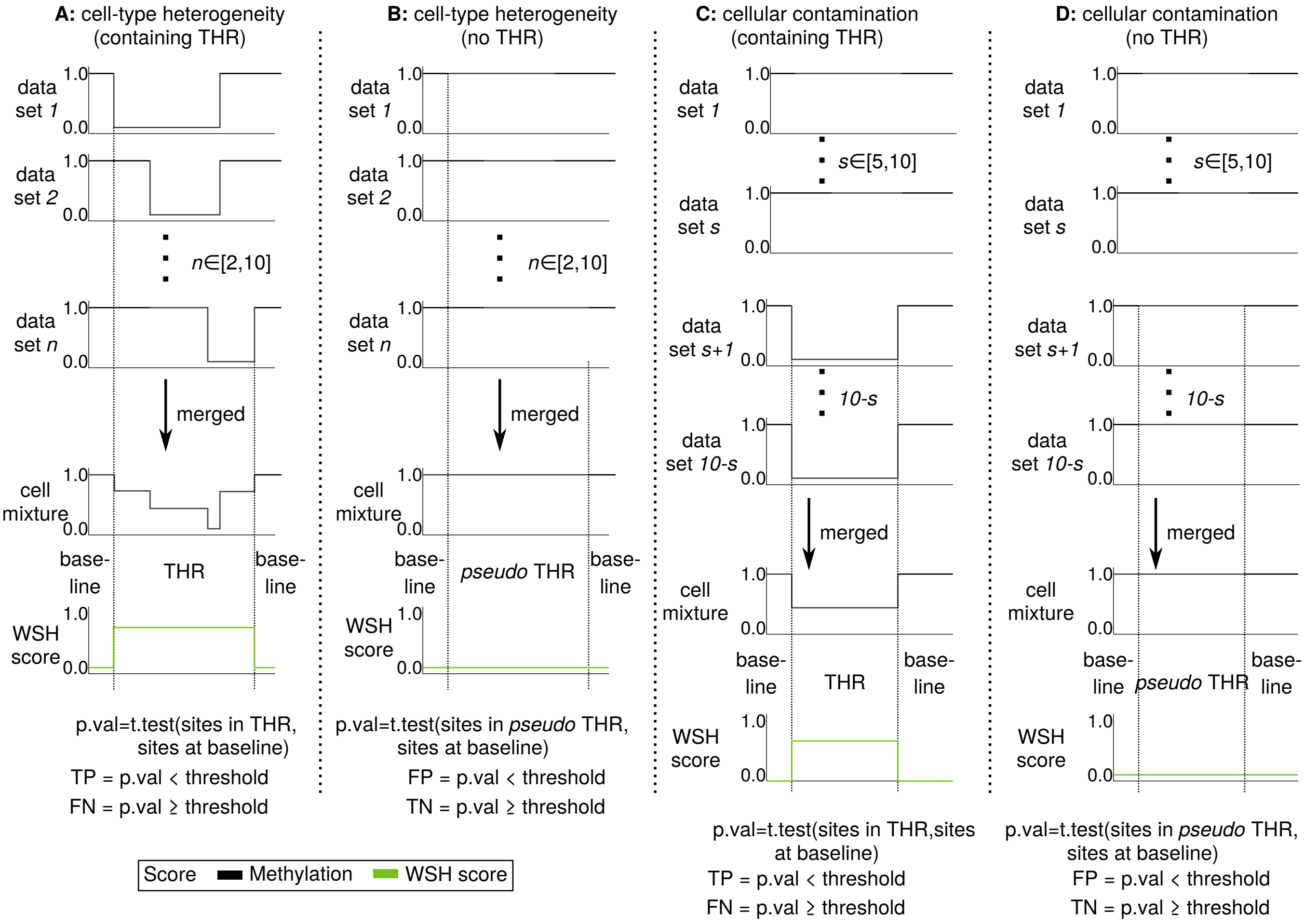

Figure S2: THR and ROC construction. For cell-type heterogeneity (**A,B**) and cellular contamination (**C,D**), a region with a THR (positive cases, **A,C**) and without a THR (negative cases, **B,D**) are shown. In total, $1,000$ of these regions were simulated with positive and negative cases chosen at random **A,B:** Between 2 and 10 datasets (representing cell types, number of cell types n) are mixed and WSH scores computed on the cellular mixture. The THR spans the union of the subregions in the individual datasets that change the methylation state. **C,D** The contaminating cell type comprises between 0% and 50% (sample purity level s). The final region comprises s times the background cell type and $10 - s$ times the contaminating cell type. A t-test determines if the sites within the THR are different from the sites with the baseline heterogeneity level. True Positives (TP) and False Negatives (FN) are computed on the positive, False Positives (FP) and True Negatives (TN) on the negatives cases. t.test: two-sided Student's t-test, p.val: p-value produced by t.test.

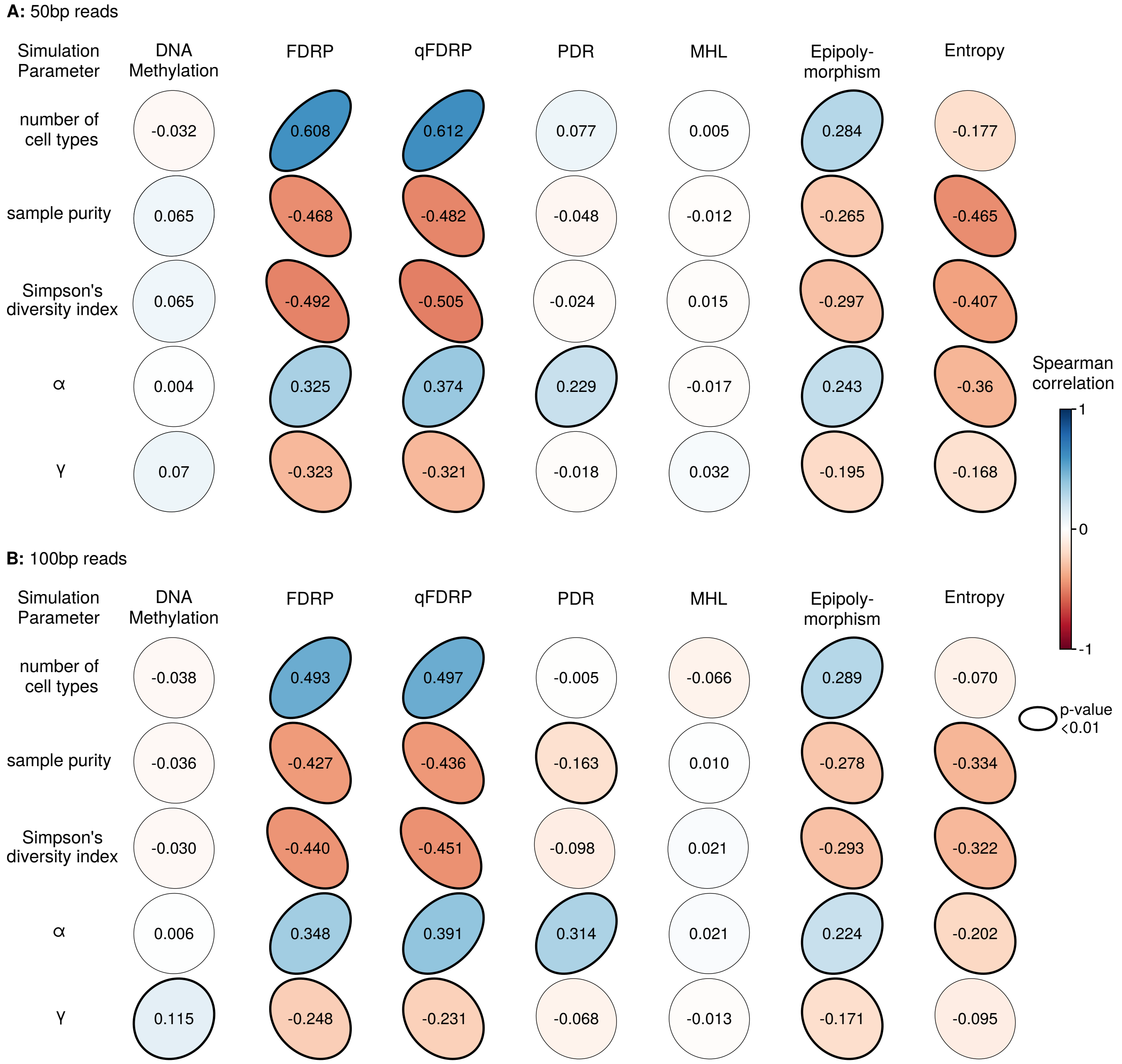

Figure S3: Spearman correlation coefficients and associated p-values between the WSH scores and the simulation parameters: number of cell types, sample purity score, Simpson's diversity index (sum of squared frequencies of the two cell types), α (stochasticity) and γ (replication) for 50 bp (**A**) and 100 bp (**B**) simulated reads. Ellipses are directed towards the upper right for positive, and to the lower right for negative correlations, respectively. The color represents the magnitude of correlation. WSH scores were averaged over all CpG positions in each of the regions and this score was correlated to the simulation parameter used for this particular region. Bold border lines indicate significant correlations.

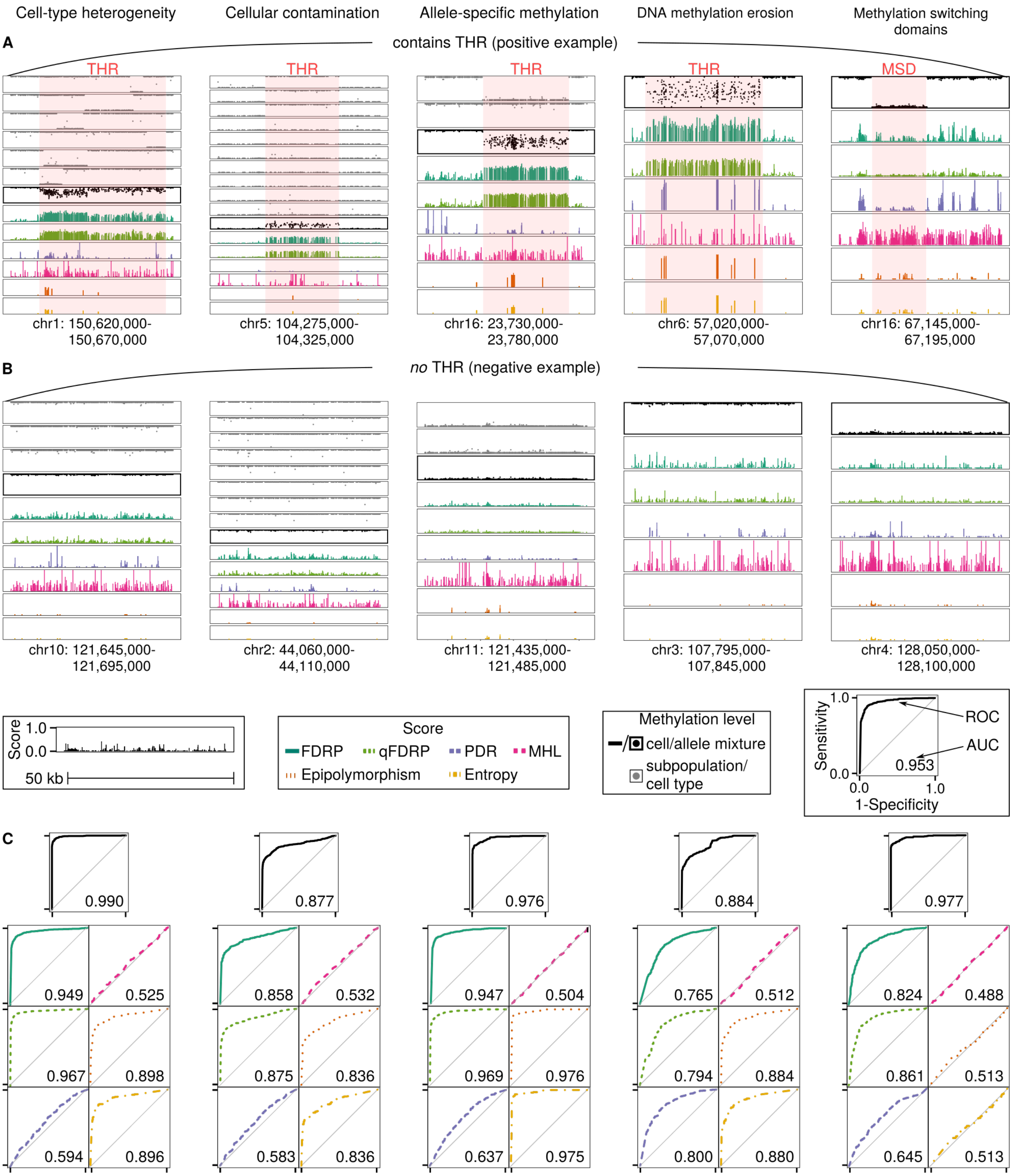

Figure S4: WSH scores in the five simulation scenarios for 100 bp reads: cell-type heterogeneity, cellular contamination, ASM, DNA methylation erosion, and methylation switching domains. A positive (**A**) and negative (**B**) example is shown as snapshot for the scores, and DNA methylation levels of single cell types (gray) and cellular mixture (black) for each scenario. $1,000$ regions of size 50 kb were simulated for each of the scenarios. **C** ROC curves represent whether the score/DNA methylation reliably differentiates truly heterogeneous regions (THRs) from the background using the p-value of a t-test.

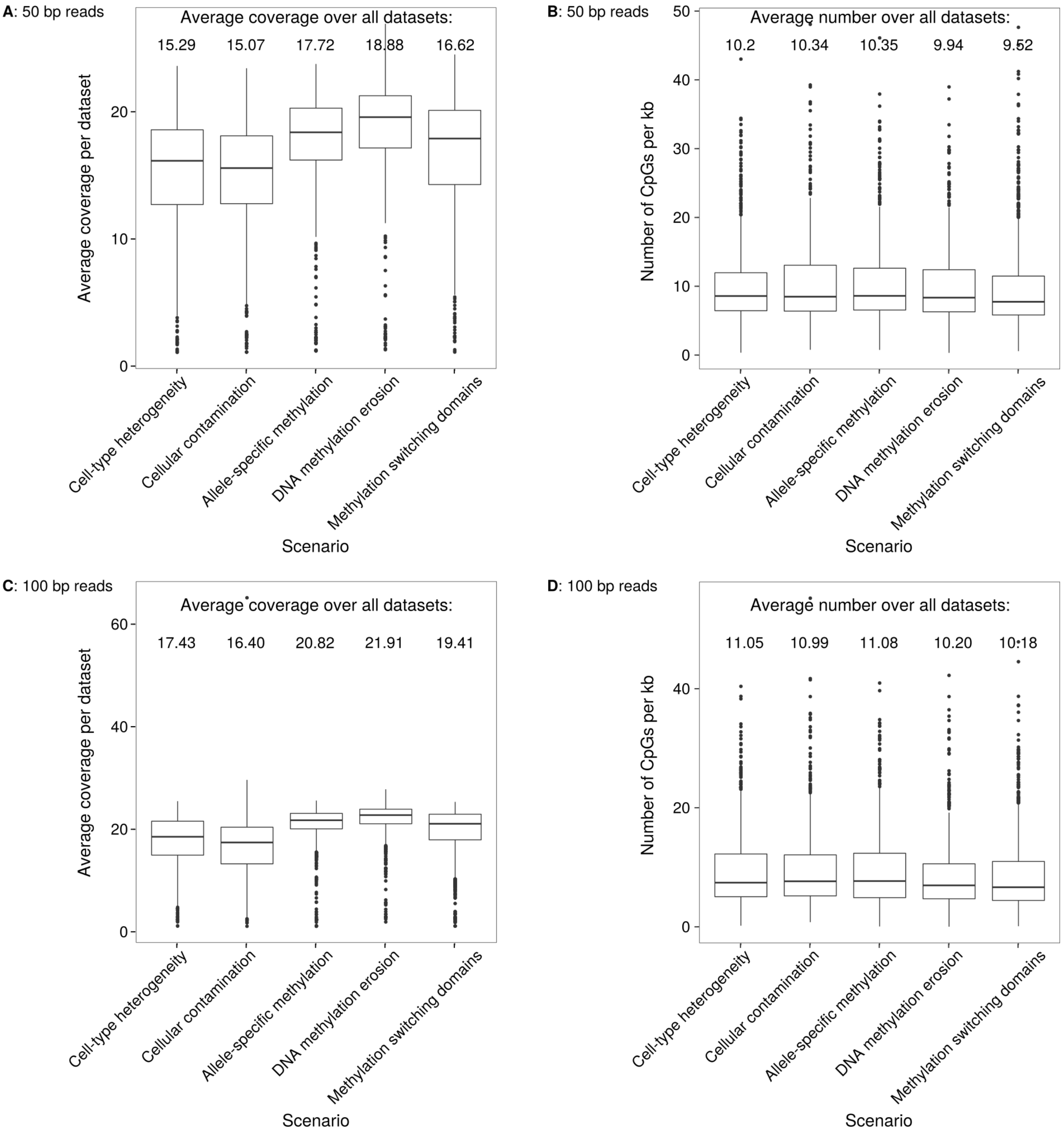

Figure S5: CpG coverage and CpG density statistics for the simulated heterogeneity scenarios. **A,C** Average coverage for all sites in the regions used for each of the heterogeneity scenarios and the average over all regions. **B,D** CpG density as the average number of CpG sites in 1kb windows for each region of the heterogeneity scenarios simulated and the average over all regions. **A,B** refer to 50 bp simulated reads and **C,D** to 100 bp read length.

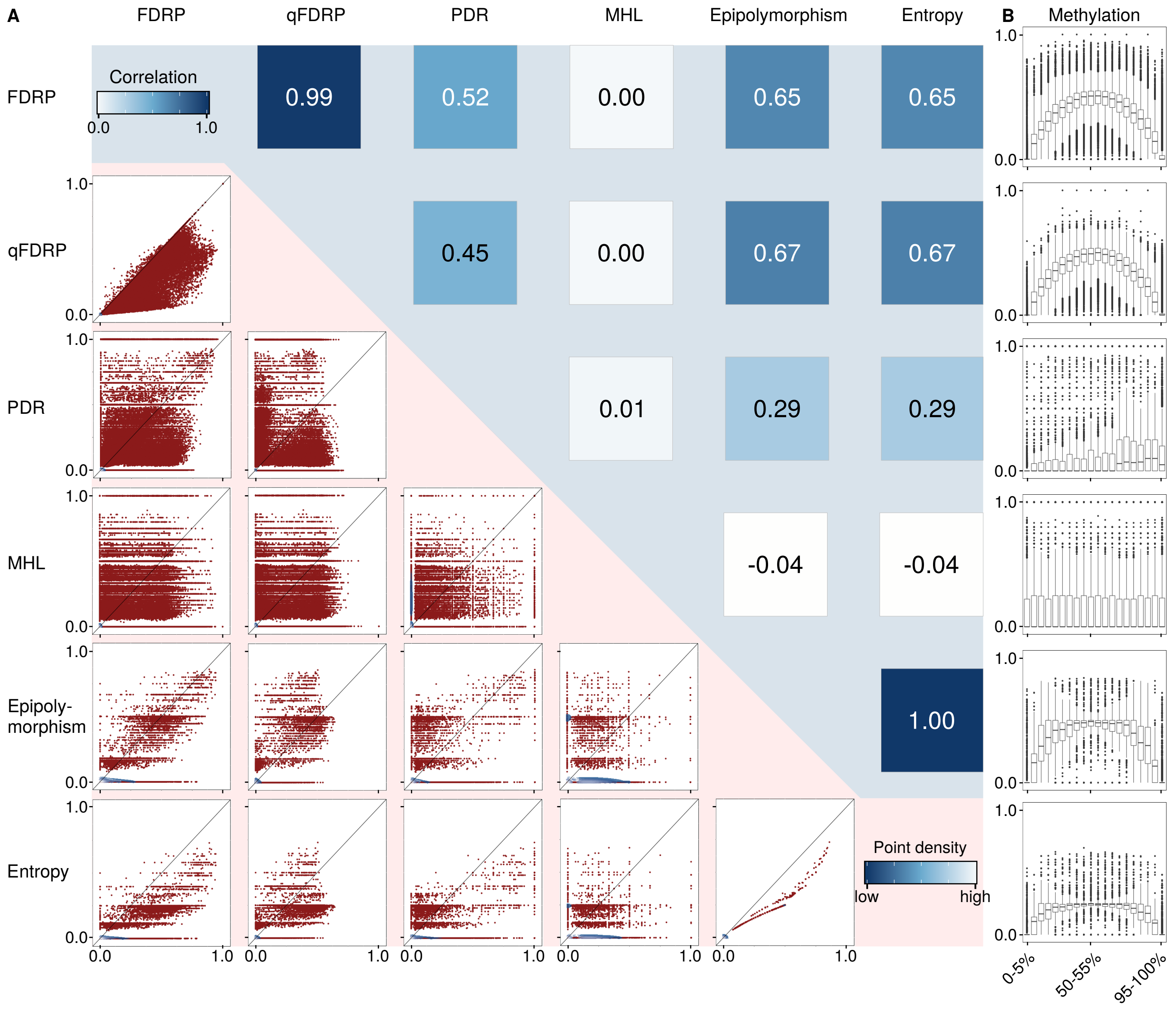

Figure S6: Inter-score comparison on simulated data (50 bp reads). **A** WSH score interconnections using all positive examples in the four heterogeneity simulation scenarios cell-type heterogeneity, cellular contamination, ASM and DNA methylation erosion. Red triangle: Scatterplots comparing WSH scores. Each red point is a CpG site or four CpG window for which both scores quantified WSH. Blue triangle: Spearman correlations between the scores. **B** Dependency on DNA methylation. DNA methylation levels were binned in steps of 5% methylation.

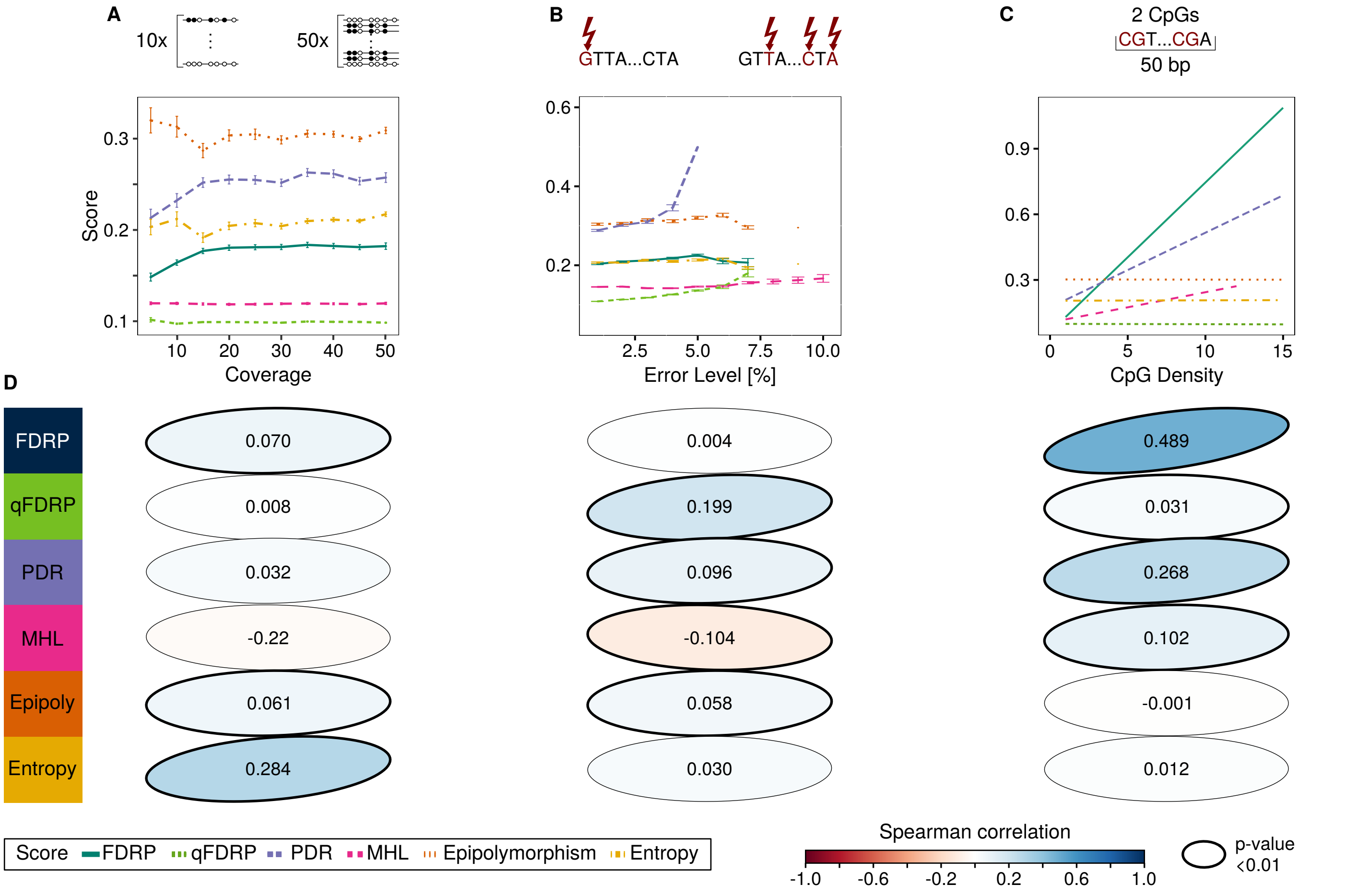

Figure S7: Effect of technical parameters on WSH scores in simulation experiments with 100 bp reads. **A** Mean WSH scores vs. mean coverage across simulated regions. The error bars indicate standard errors. **B** Mean WSH scores and standard errors vs. simulated sequencing error level in percent. **C** Least squares regression line for the number of CpGs in a 50 bp window (CpG density) and the average WSH score in this window. **D** Spearman correlations to technical parameters are shown with ellipses that are oriented towards the upper right for positive, and to the lower right for negative correlations, respectively. The color represents the magnitude of correlation and significant correlations (p-value lower than 0.01) are indicated by bold border lines.

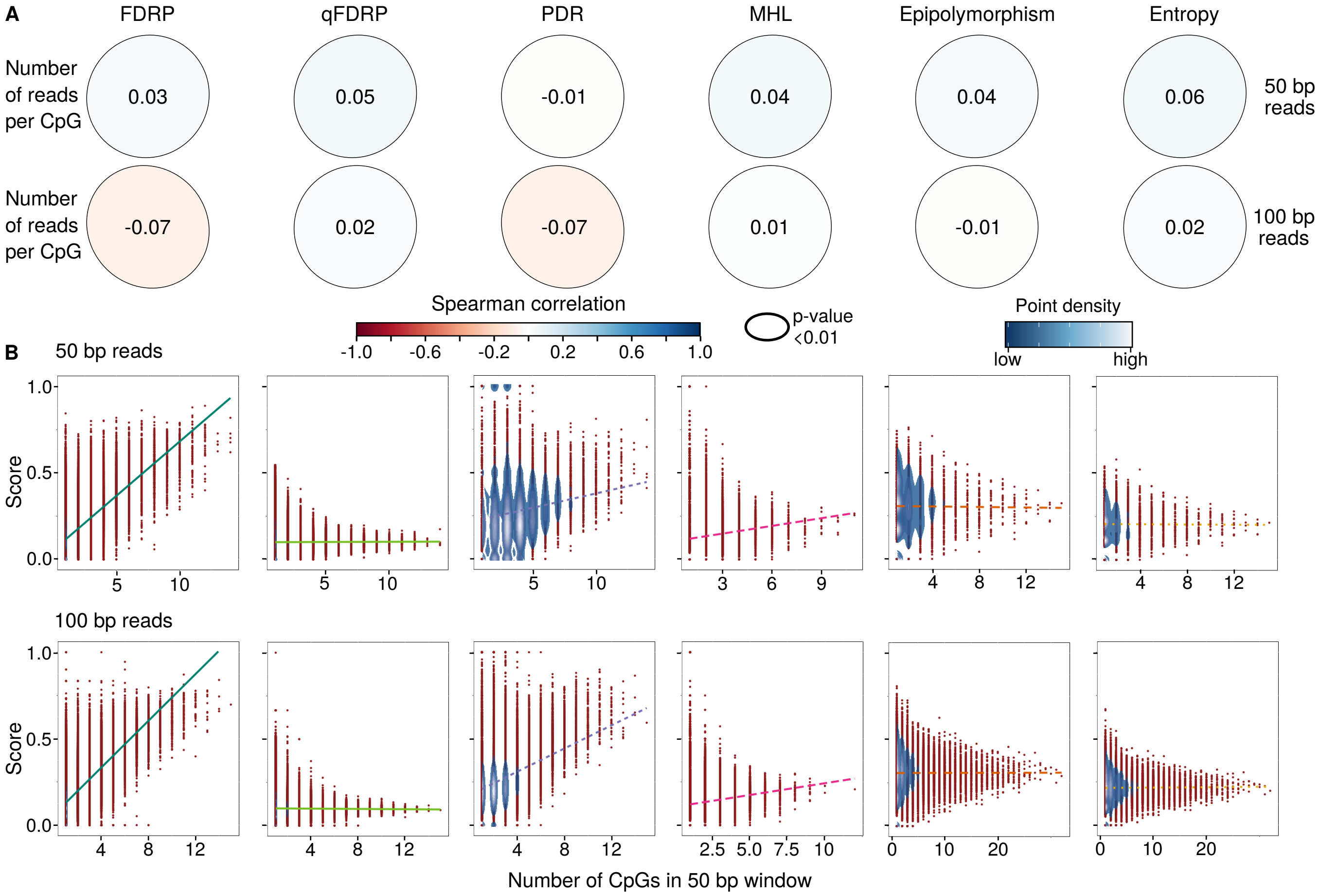

Figure S8: WSH scores dependencies to coverage and CpG density. **A** Spearman's rank correlation between the site-wise number of reads (coverage) and the WSH scores. **B** Connections between number of CpG sites in a 50 bp window and the average WSH score. Results were calculated for $1,000$ random regions with $25,000$ reads simulated, and computed for 50 bp and 100 bp reads separately. Lighter blue indicates higher point density and the solid line represents the least squares regression line.

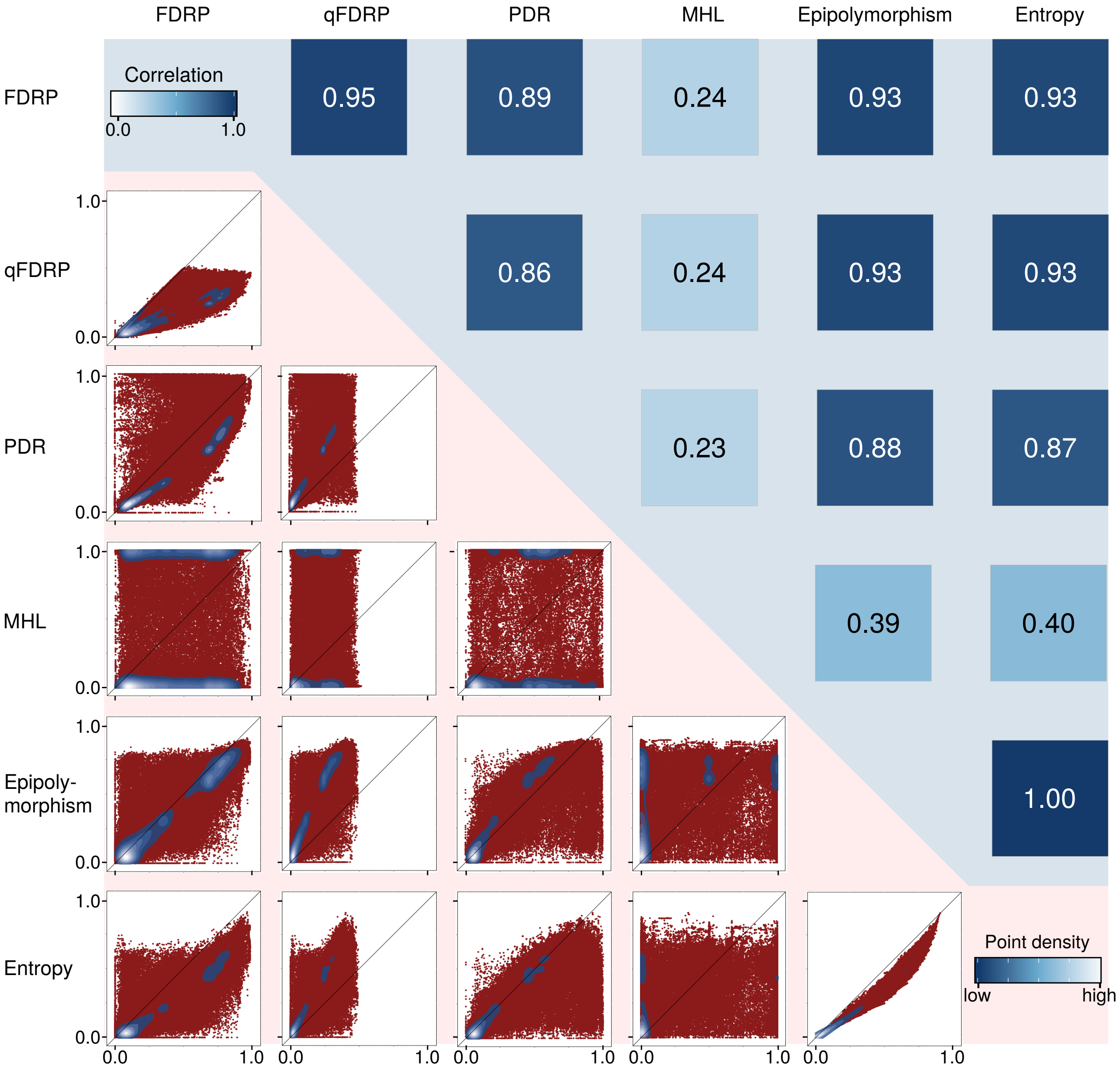

Figure S9: Inter-score comparison on the blood cohort dataset. Values were first averaged over all samples to produce a score at each region and then those scores were plotted against each other (red triangle) or the Spearman's rank correlation was computed. Lighter blue represents higher point density.

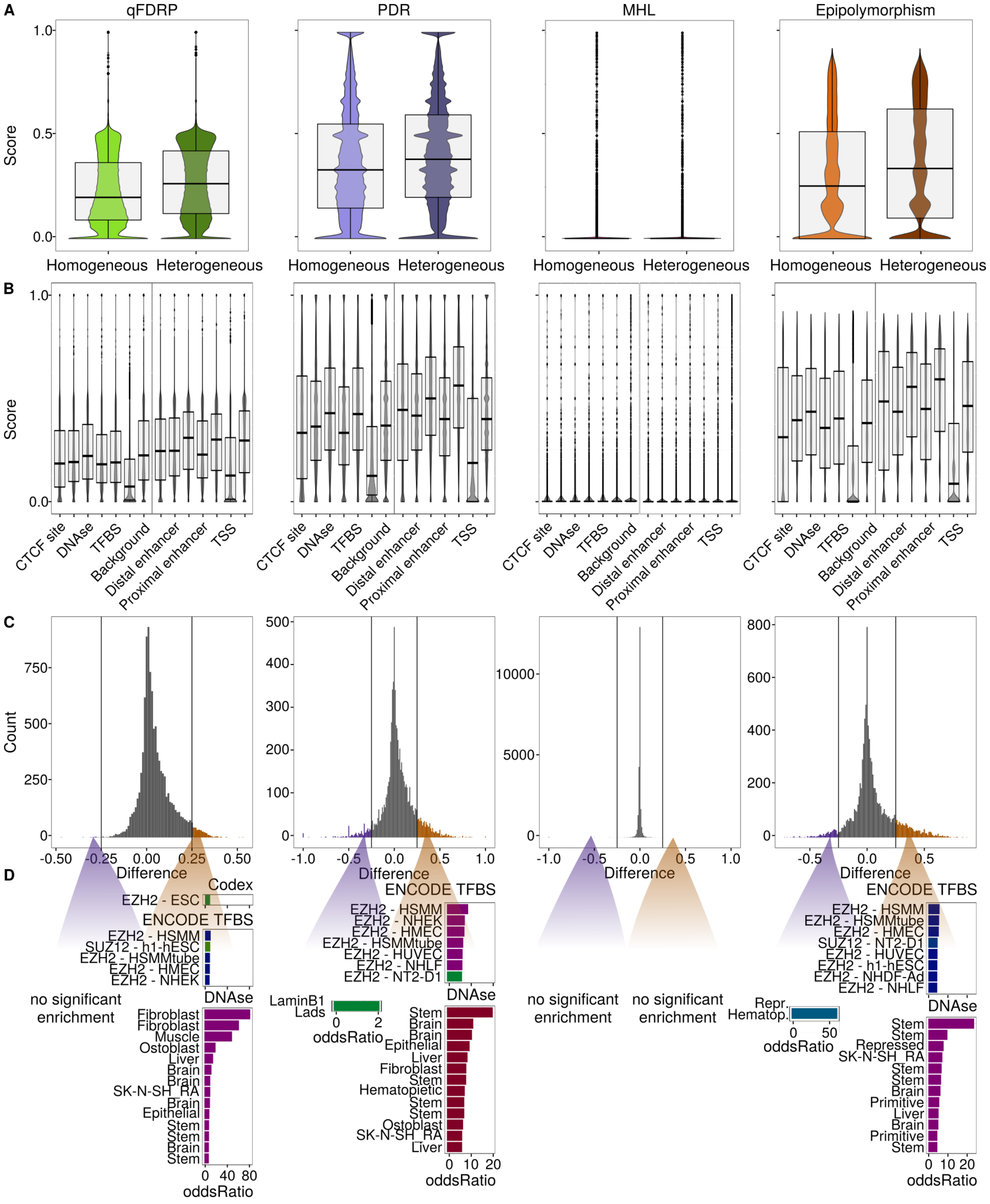

Figure S10: WSH scores in homogeneous and heterogeneous hybrid samples comprising similar (hepatocytes) or distinct (hepatocytes and liver cel lines) samples from the DEEP project. **A** Genome-wide distribution of the WSH scores qFDRP, PDR, MHL and Epipolymorphism for the homogeneous and the heterogeneous sample separately. **B** Stratification of genomic locations according to the Ensembl Regulatory Build and distributions within each of the classes defined, shown for the homogeneous (left) and the heterogeneous (right) hybrid sample. **C** Histogram of WSH score differences aggregated over promoters (i.e. region 500 bp downstream and 1.5 kb upstream of the TSS) between the heterogeneous and homogeneous sample. We set 0.25 as threshold and performed LOLA enrichment analysis (**D**) for the promoters that had higher heterogeneity in the homogeneous (purple) or the heterogeneous (brown) sample.

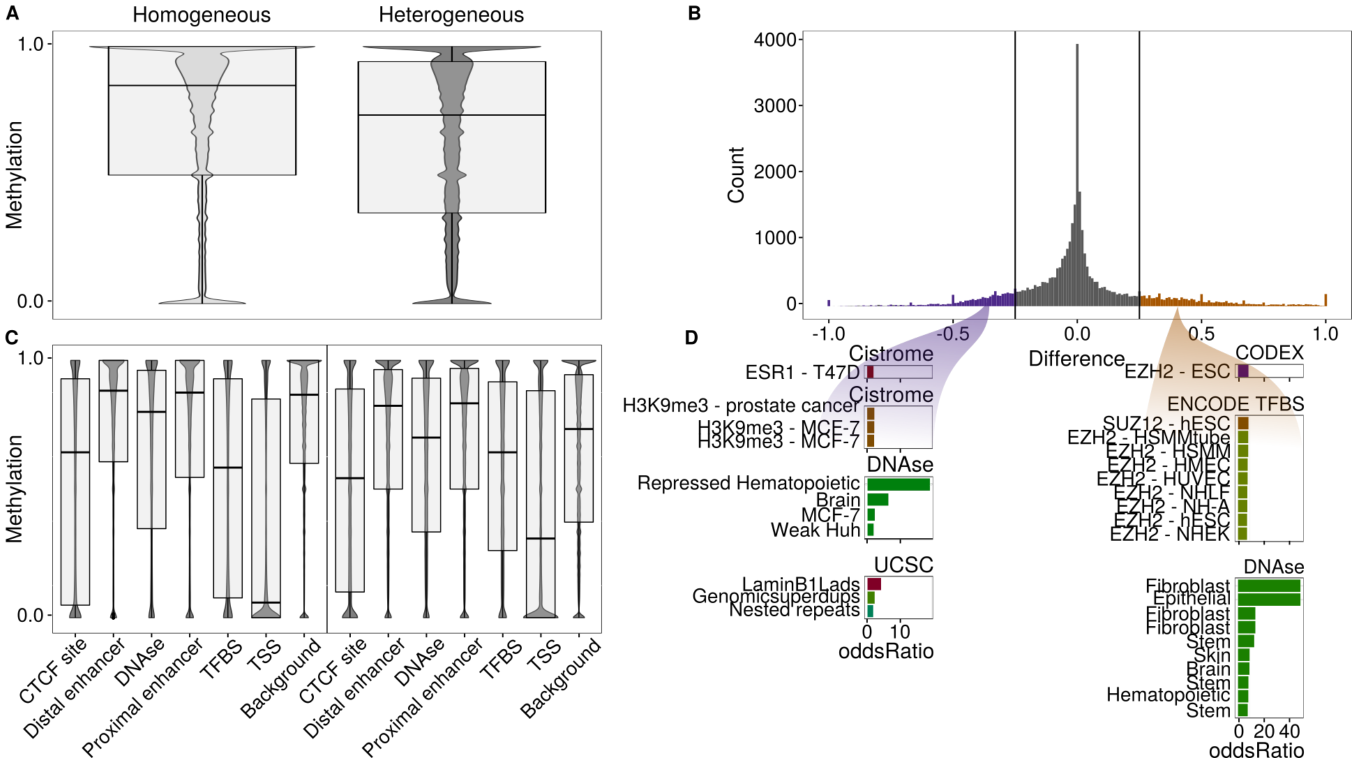

Figure S11: DNA methylation in DEEP hybrid samples. **A** Genome-wide distribution of DNA methylation for the homogeneous and heterogeneous sample separately. **B** Histogram of DNA methylation differences aggregated over promoters (i.e. region 500 bp downstream and 1.5 kb upstream of the TSS of annotated Ensembl genes) between the heterogeneous and the homogeneous sample. **C** Stratification of DNA methylation according to the Ensembl Regulatory Build for the homogeneous (left) and the heterogeneous (right) sample. **D** LOLA enrichment analysis for the promoters that had higher DNA methylation (threshold 0.25) in the homogeneous (purple) or the heterogeneous (brown) sample.

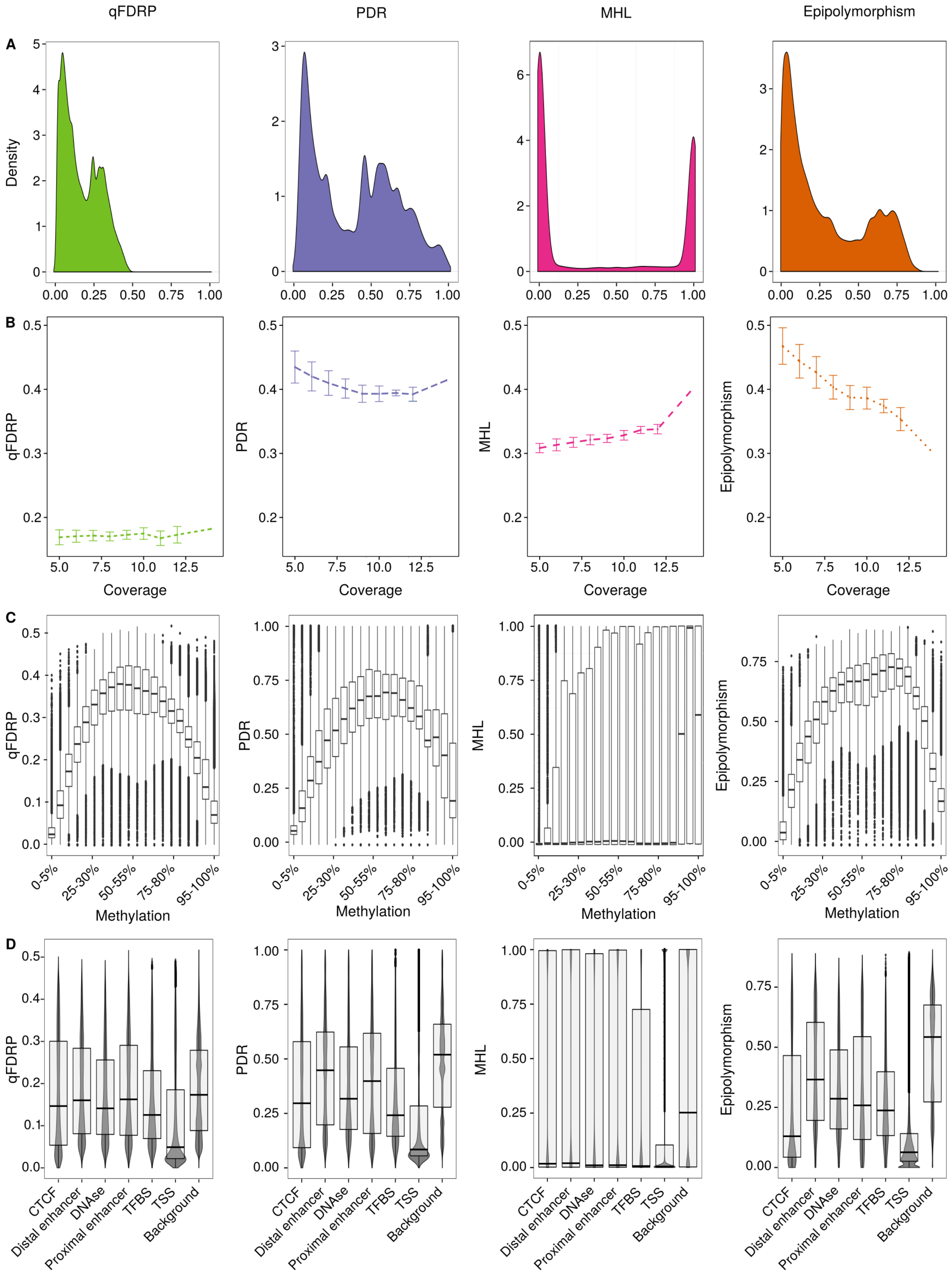

Figure S12: WSH scores (qFDRP, PDR, MHL and Epipolymorphism) in the blood dataset. **A** Genome wide-distribution of WSH scores after computing site-wise averages over all samples. **B** Per-sample average WSH score vs. per-sample average coverage. **C** Site-wise average WSH score over all samples vs. DNA methylation stratified into 20 classes of size 5 each. **D** Distribution of site-wise average WSH scores over all samples across the Ensembl regulatory build region types.

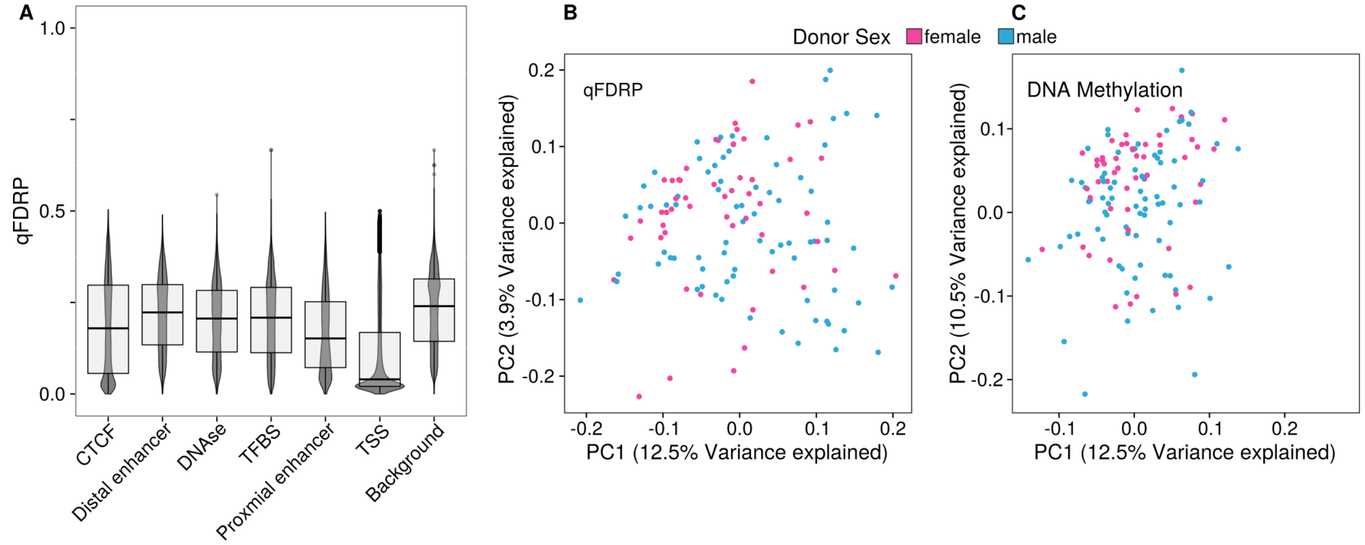

Figure S13: qFDRP scores in Ewing tissue samples. **A** Distribution of CpG-wise average qFDRP scores over all samples according to the regions defined by the Ensembl regulatory build. **B** PCA of qFDRP scores aggregated along Ensembl gene bodies. Different colors indicate different donor sexes. **C** PCA plot of DNA methylation levels similar to **B**.

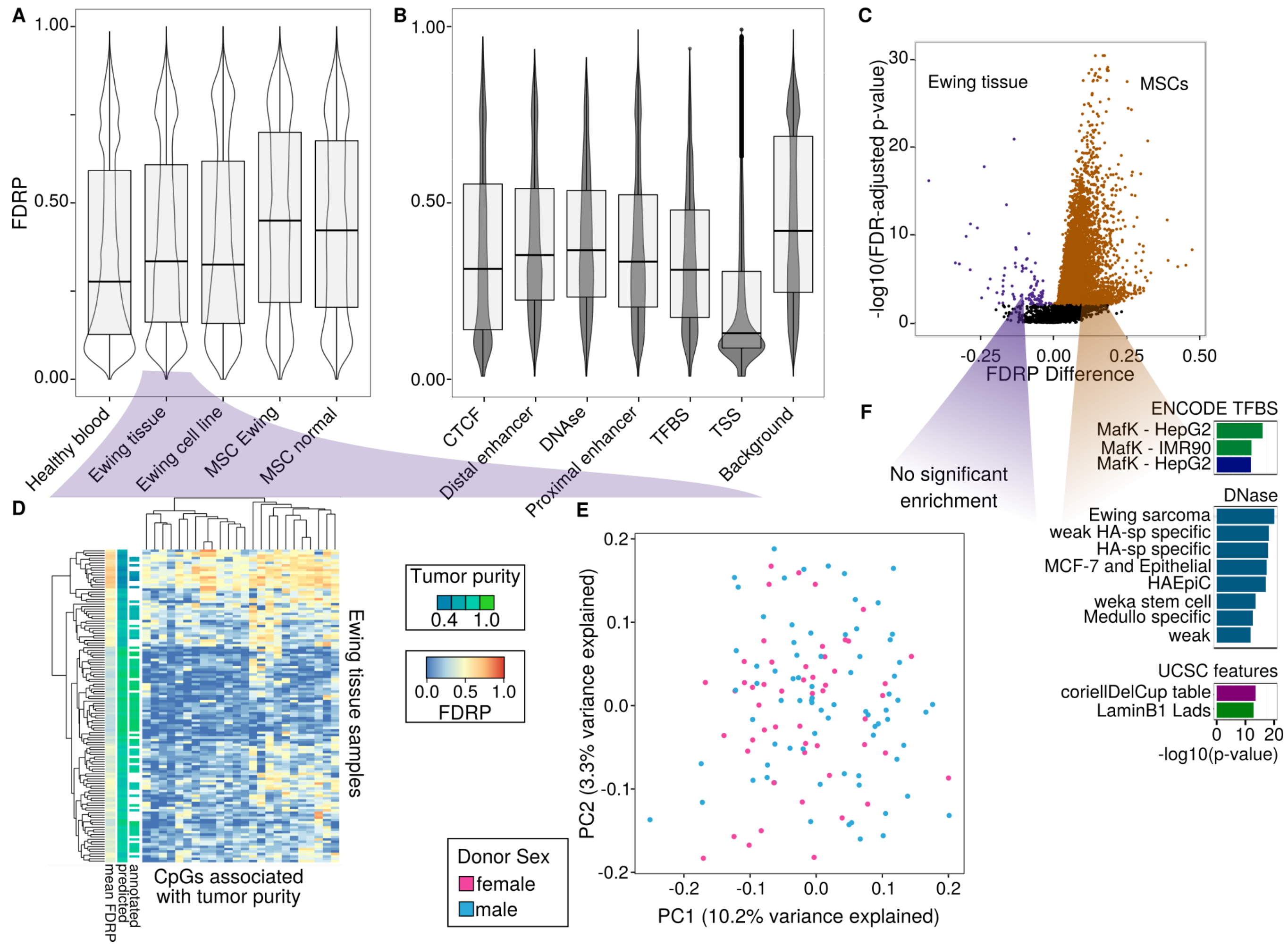

Figure S14: FDRP scores in Ewing dataset **A** Combined box- and violin plots of different sample groups. The leftmost group are samples from the blood cohort, while the others are different sample categories within the Ewing dataset. **B** Distribution of mean site-wise FDRP scores according to the Ensembl regulatory build for the Ewing dataset only. **C** Volcano plot of FDRP values aggregated over gene bodies in Ewing tissue samples versus MSCs. P-values were calculated with limma and positive values on the x-axis indicate higher WSH in MSCs. Marked in brown/purple are the genes that had FDR-adjusted p-value lower than 0.01. **D** Heatmap (complete linkage, Euclidean distance) of FDRP scores at sites linked to tumor purity for Ewing tissue samples only, excluding formalin-fixed and paraffin-embedded (FFPE) samples. Red represents high, while blue represents lower heterogeneity and sample colors visualize predicted and annotated tumor purity levels, respectively, and the average FDRP score over the selected sites. **E** PCA of FDRP scores aggregated along genes for Ewing tissue samples only. **F** LOLA enrichment analysis of the hyper-heterogeneous genes in the MSCs vs. Ewing tissue samples. Histograms indicate the negative logarithm of the p-value computed by LOLA.

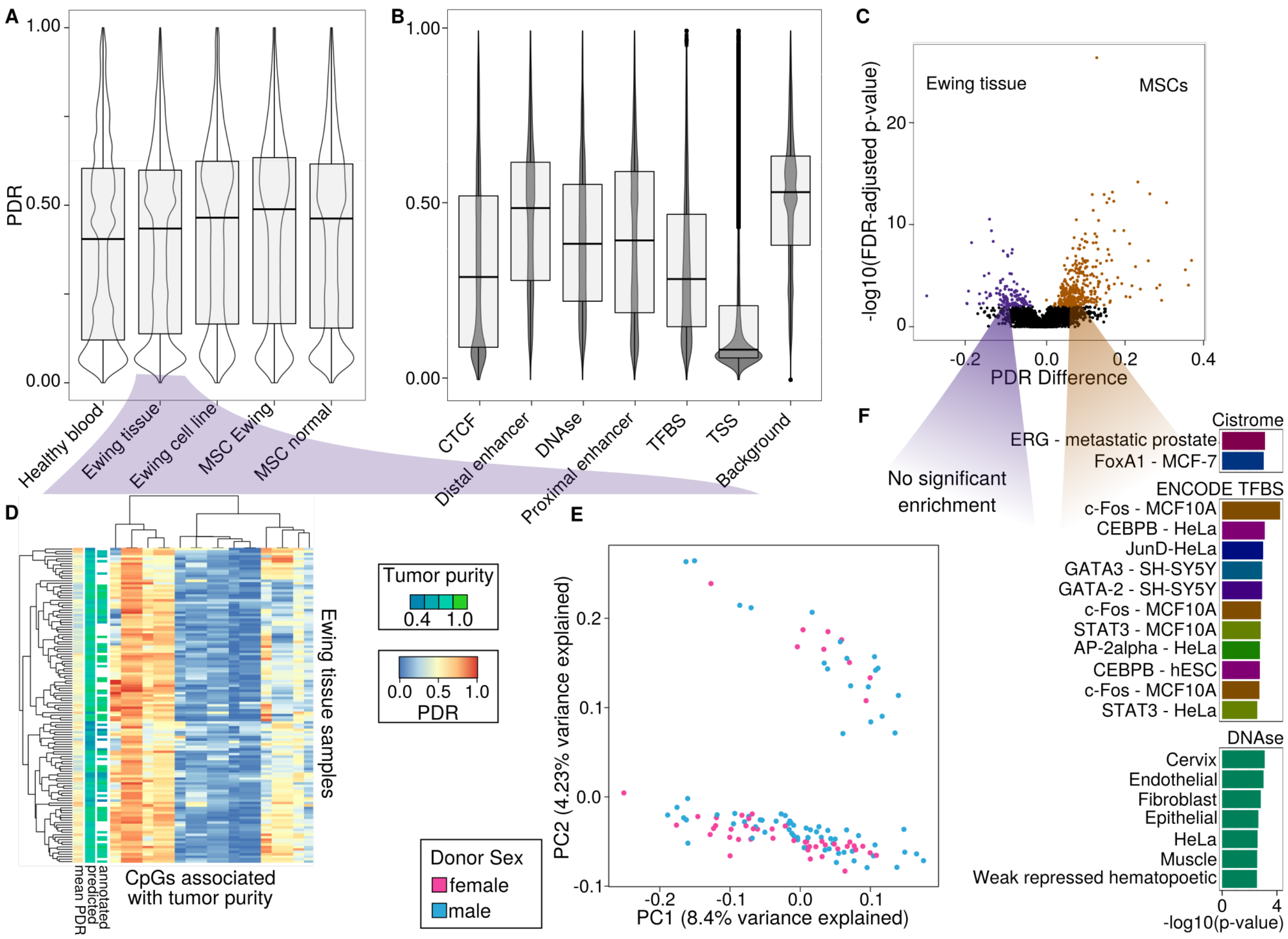

Figure S15: PDR scores in Ewing dataset **A** Combined box- and violin plots of different sample groups. The leftmost group are samples from the blood cohort, while the others are different sample categories within the Ewing dataset. **B** Distribution of mean site-wise PDR scores according to the Ensembl regulatory build for the Ewing dataset only. **C** Volcano plot of PDR values aggregated over gene bodies in Ewing tissue samples versus MSCs. P-values were calculated with limma and positive values on the x-axis indicate higher WSH in MSCs. Marked in brown/purple are the genes that had FDR-adjusted p-value lower than 0.01. **D** Heatmap (complete linkage, Euclidean distance) of PDR scores at sites linked to tumor purity for Ewing tissue samples only, excluding FFPE samples. Red represents high, while blue represents lower heterogeneity and sample colors visualize predicted and annotated tumor purity levels, respectively, and the average PDR score over the selected sites. **E** PCA of PDR scores aggregated along genes for Ewing tissue samples only. **F** LOLA enrichment analysis of the hyper-heterogeneous genes in the MSCs vs. Ewing tissue samples. Histograms indicate the negative logarithm of the p-value computed by LOLA.

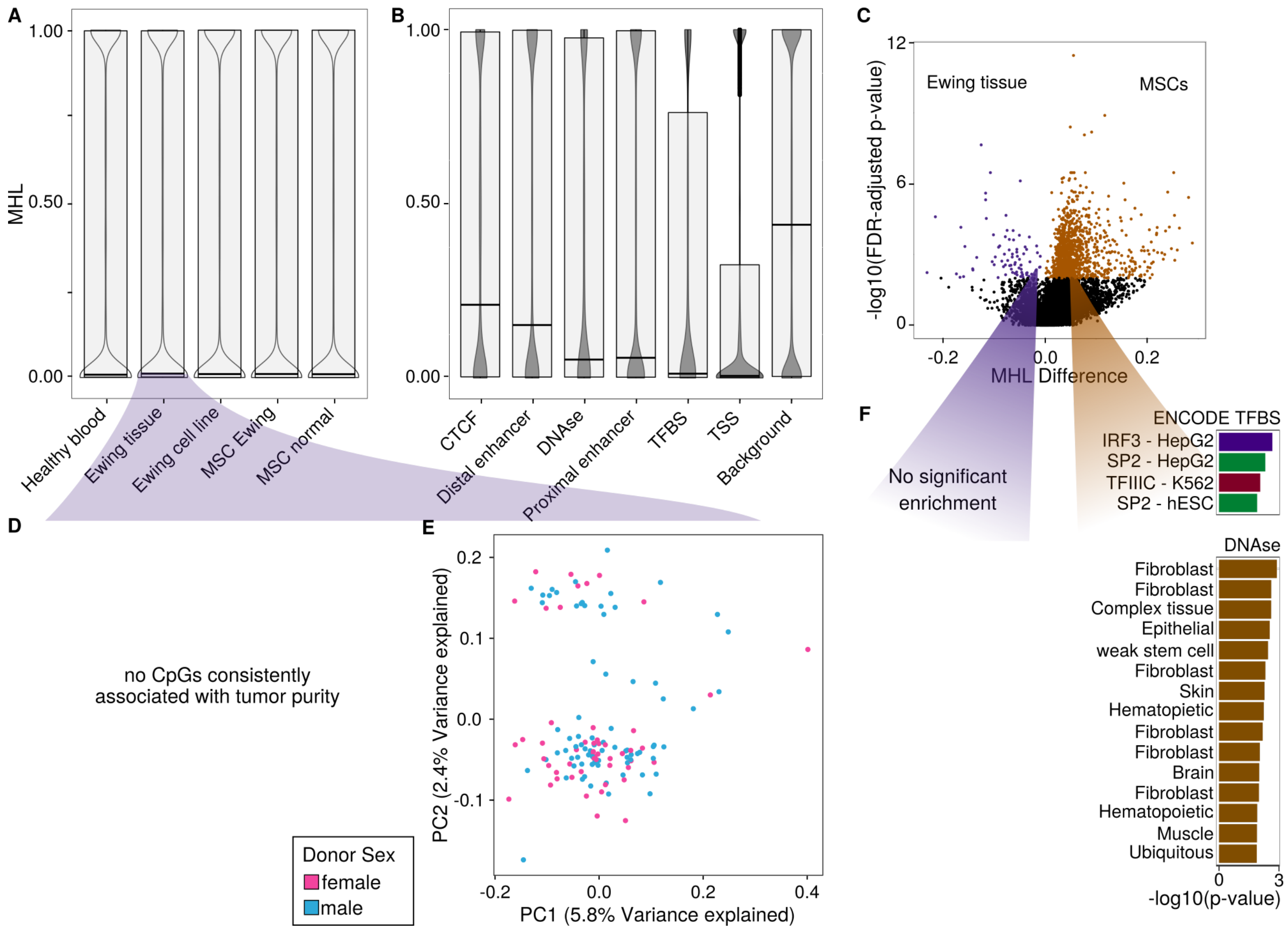

Figure S16: MHL scores in Ewing dataset **A** Combined box- and violin plots of different sample groups. The leftmost group are samples from the blood cohort, while the others are different sample categories within the Ewing dataset. **B** Distribution of mean site-wise MHL scores according to the Ensembl regulatory build for the Ewing dataset only. **C** Volcano plot of MHL values aggregated over gene bodies in Ewing tissue samples versus MSCs. P-values were calculated with limma and positive values on the x-axis indicate higher WSH in MSCs. Marked in brown/purple are the genes that had FDR-adjusted p-value lower than 0.01. **D** No MHL levels at CpGs were consistently linked to tumor purity. **E** PCA of MHL scores aggregated along genes for Ewing tissue samples only, without FFPE samples. **F** LOLA enrichment analysis of the hyper-heterogeneous genes in the MSCs vs. Ewing tissue samples. Histograms indicate the negative logarithm of the p-value computed by LOLA.

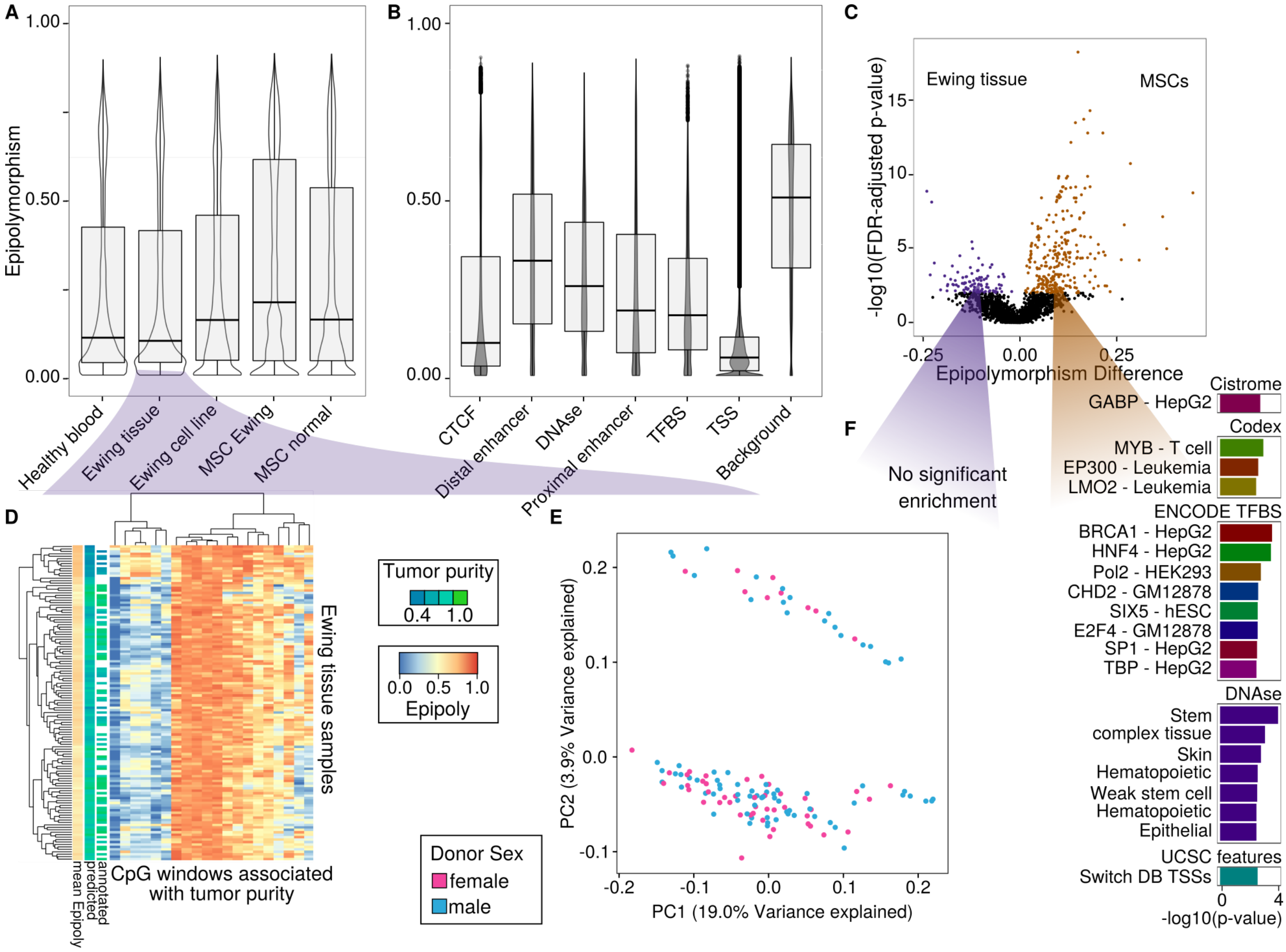

Figure S17: Epipolymorphism scores in Ewing dataset **A** Combined box- and violin plots of different sample groups. The leftmost group are samples from the blood cohort, while the others are different sample categories within the Ewing dataset. **B** Distribution of mean window-wise Epipolymorphism scores according to the Ensembl regulatory build for the Ewing dataset only. **C** Volcano plot of Epipolymorphism values aggregated over gene bodies in Ewing tissue samples versus MSCs. P-values were calculated with limma and positive values on the x-axis indicate higher WSH in MSCs. Marked in brown/purple are the genes that had FDR-adjusted p-value lower than 0.01. **D** Heatmap (complete linkage, Euclidean distance) of Epipolymorphism scores at CpG windows linked to tumor purity for Ewing tissue samples only, excluding FFPE samples. Red represents high, while blue represents lower WSH and sample colors visualize predicted and annotated tumor purity levels, respectively, and the average Epipolymorphism score over the selected sites. **E** PCA of Epipolymorphism scores aggregated along genes for Ewing tissue samples only. **F** LOLA enrichment analysis of the hyper-heterogeneous genes in the MSCs vs. Ewing tissue samples. Histograms indicate the negative logarithm of the p-value computed by LOLA.

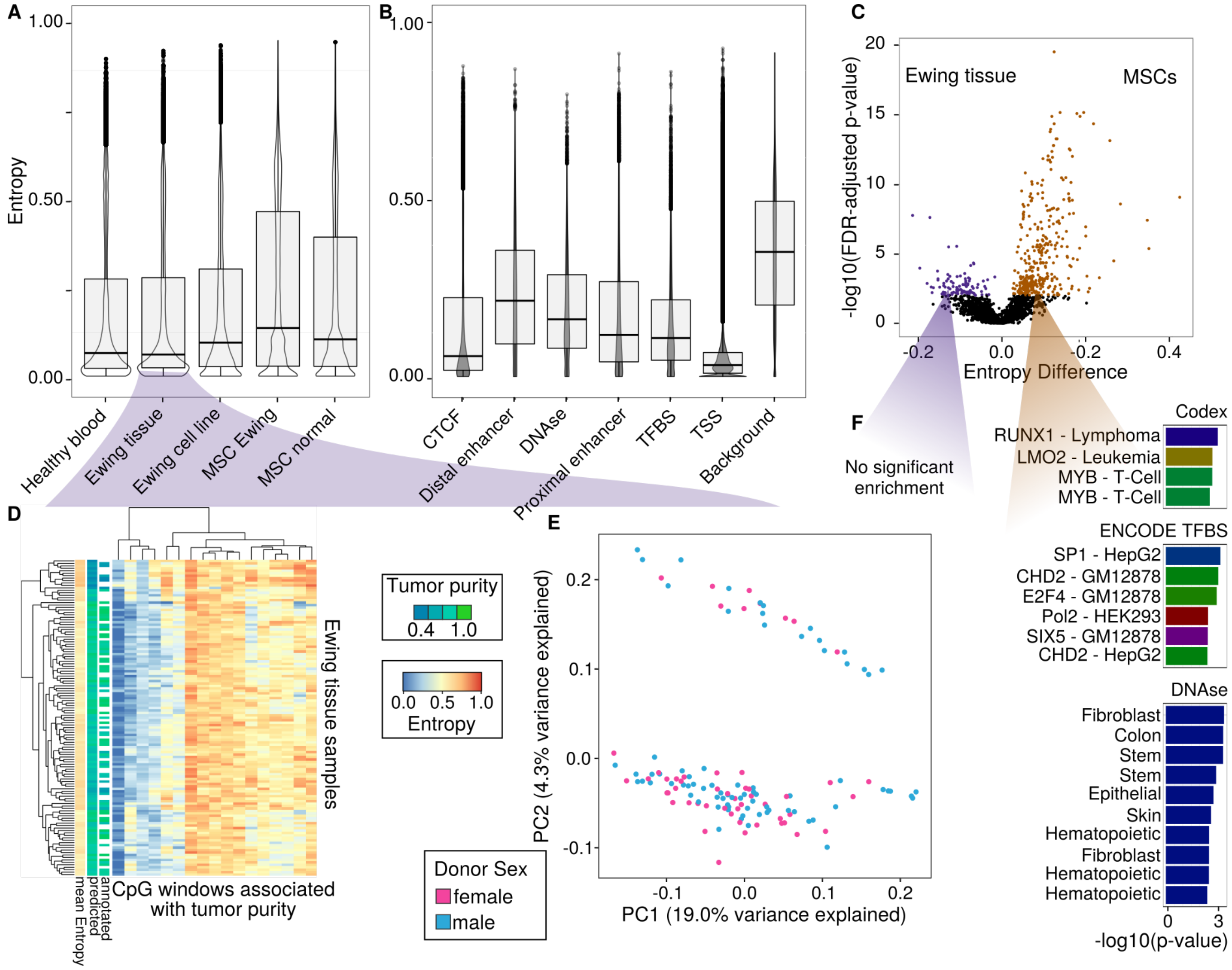

Figure S18: Entropy scores in Ewing dataset **A** Combined box- and violin plots of different sample groups. The leftmost group are samples from the blood cohort, while the others are different sample categories within the Ewing dataset. **B** Distribution of mean window-wise Entropy scores according to the Ensembl regulatory build for the Ewing dataset only. **C** Volcano plot of Entropy values aggregated over gene bodies in Ewing tissue samples versus MSCs. P-values were calculated with limma and positive values on the x-axis indicate higher WSH in MSCs. Marked in brown/purple are the genes that had FDR-adjusted p-value lower than 0.01. **D** Heatmap (complete linkage, Euclidean distance) of Entropy scores at CpG windows linked to tumor purity for Ewing tissue samples only, excluding FFPE samples. Red represents high, while blue represents lower WSH and sample colors visualize predicted and annotated tumor purity levels, respectively, and the average Entropy score over the selected sites. **E** PCA of Entropy scores aggregated along genes for Ewing tissue samples only. **F** LOLA enrichment analysis of the hyper-heterogeneous genes in the MSCs vs. Ewing tissue samples. Histograms indicate the negative logarithm of the p-value computed by LOLA.

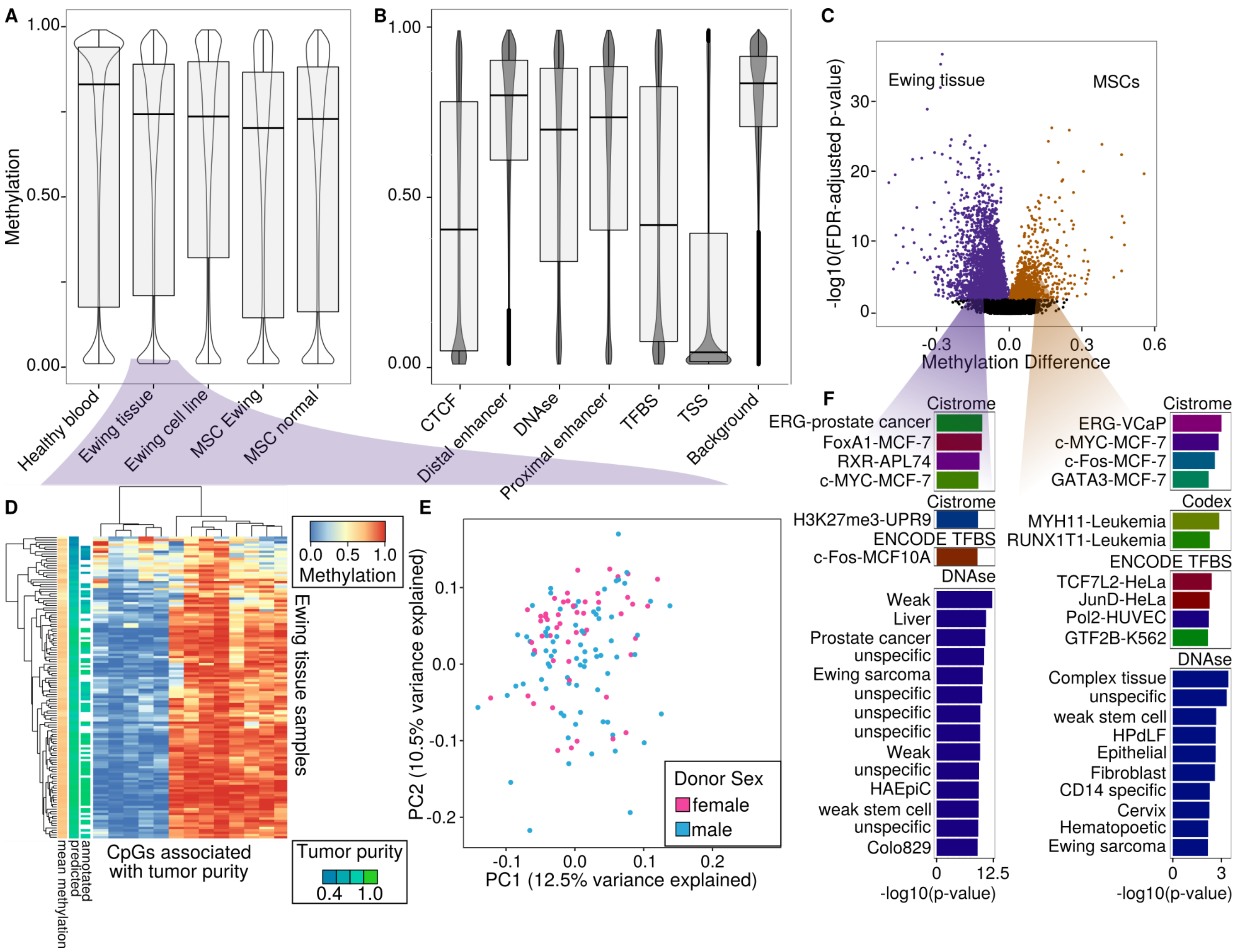

Figure S19: DNA methylation levels in Ewing dataset **A** Combined box- and violin plots of different sample groups. The leftmost group are samples from the blood cohort, while the others are different sample categories within the Ewing dataset. **B** Distribution of mean site-wise DNA methylation levels according to the Ensembl regulatory build for the Ewing dataset only. **C** Volcano plot of DNA methylation levels aggregated over gene bodies in Ewing tissue samples versus MSCs. P-values were calculated with limma and positive values on the x-axis indicate higher DNA methylation in MSCs. Marked in brown/purple are the points that had FDR-adjusted p-value lower than 0.01. **D** Heatmap (complete linkage, Euclidean distance) of DNA methylation levels at sites linked to tumor purity for Ewing tissue samples only, excluding FFPE samples. Red represents high, while blue represents lower DNA methylation level and sample colors visualize predicted and annotated tumor purity levels, respectively, and the average DNA methylation level over the selected sites. **E** PCA of DNA methylation levels aggregated along genes for Ewing tissue samples only. **F** LOLA enrichment analysis of the hypo- and hyper-methylated genes in the MSCs vs. Ewing tissue samples. Histograms indicate the negative logarithm of the p-value computed by LOLA.

Supplementary Tables

Table S1: Examples of read configurations and resulting WSH scores.

Reads	FDRP	qFDRP	PDR	Epipolymorphism	Entropy	MHL
	$\frac{\text{\#discordant rp}}{\text{\#all rp}}$	$\frac{\sum_{rp} df_{rp}}{\text{\#all rp}}$	$\frac{\text{\#discordant reads}}{\text{\#all reads}}$	$1 - \sum_{i=1}^{16} p_i^2$	$-\frac{1}{4} \sum_{i=1}^{16} p_i * \log_2 p_i$	$\frac{\sum_{i=1}^{l} w_i * P(MH_i)}{\sum_{i=1}^{l} w_i}$
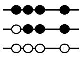	$\frac{3}{3} = 1$	$\frac{\frac{1}{4}+1+\frac{3}{4}}{3} = \frac{2}{3}$	$\frac{1}{3}$	$1 - 3 * (\frac{1}{3})^2 = \frac{2}{3}$	$-\frac{1}{4} * \log_2 \frac{1}{3} = 0.396$	$\frac{1*\frac{7}{12}+2*\frac{5}{9}+3*\frac{3}{6}+4*\frac{1}{3}}{1+2+3+4} = 0.4528$
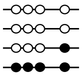	$\frac{5}{6}$	$\frac{\frac{1}{4}+1+\frac{1}{4}+1+\frac{3}{4}}{6} = \frac{13}{24}$	$\frac{1}{4}$	$1 - (\frac{1}{2})^2 - 2 * (\frac{1}{4})^2 = \frac{5}{8}$	$-\frac{1}{4} * (\frac{1}{2} \log_2 \frac{1}{2} + \frac{1}{2} \log_2 \frac{1}{4}) = 0.375$	$\frac{1*\frac{5}{16}+2*\frac{3}{12}+3*\frac{2}{8}+4*\frac{1}{4}}{1+2+3+4} = 0.2562$
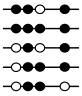	$\frac{10}{10} = 1$	$\frac{\frac{1}{4}+\frac{1}{4}+\frac{2}{4}+\frac{3}{4}}{10} + \frac{\frac{2}{4}+\frac{1}{4}+\frac{2}{4}}{10} + \frac{\frac{1}{4}+\frac{2}{4}}{10} + \frac{\frac{1}{4}}{10} = \frac{16}{40} = \frac{2}{5}$	$\frac{4}{5}$	$1 - 5 * (\frac{1}{5})^2 = \frac{4}{5}$	$-\frac{1}{4} * \log_2 \frac{1}{5} = 0.58$	$\frac{1*\frac{14}{20}+2*\frac{7}{15}+3*\frac{3}{10}+4*\frac{1}{5}}{1+2+3+4} = \frac{1}{3}$

Table S2: Overview of simulation parameters used to generate the simulated datasets. See Figure S1 for a schematic of the heterogeneity scenarios described.

	Heterogeneity scenarios					Technical biases		
**Scenario**	**Cell-type heterogeneity**	**Cellular contamination**	**Allele-specific methylation**	**DNA methylation erosion**	**Methylation switching domains**	**Coverage dependence**	**Read length dependence**	**Error dependence**
**Total number of regions**	1,000	1,000	1,000	1,000	1,000	10,000	12,000	10,000
**Size of regions**	50 kb	50 kb	50 kb	50 kb	50 kb	50 kb	50 kb	50 kb
**Number of reads generated per region**	25,000	25,000	25,000	25,000	25,000	varying	varying	25,000
`--CG_conversion`	100/0	100/0	100/0	varying	100/0	95/5	95/5	95/5
**Read length**	50 bp	50 bp	50 bp	50 bp	50 bp	50 bp	varying	50 bp
**Error level**	1%	1%	1%	1%	1%	0%	0%	varying
**Phred Quality**	40	40	40	40	40	40	40	40
`--CH_conversion`	77	77	77	77	77	77	77	77
**Test statistic employed**	t-test/ROC	t-test/ROC	t-test/ROC	t-test/ROC	(negative) t-test/ROC	correlation test	correlation test	correlation test

Table S3: Confusion matrices for the five simulation scenarios (**A,F** cell type heterogeneity, **B,G** cellular contamination, **C,H** allele-specific methylation, **D,I** DNA methylation erosion, **E,J** Methylation switching domains) for all WSH scores and the average DNA methylation level (DNAm) shown separately for 50 bp (**A-E**) and 100 bp read length (**F-J**). For the confusion matrices, a 0.01 was employed as the p-value cutoff to determine if the score detects the THR.

Score	True State	**A:** Cell-type heterogeneity		**B:** Cellular contamination		**C:** Allele-specific methylation		**D:** DNA methylation erosion		**E:** Methylation switching domains	
		THR	no THR	THR	no THR	THR	no THR	THR	no THR	THR	no THR
DNAm	THR	466	25	340	105	481	31	408	71	496	20
DNAm	no THR	62	355	73	366	84	382	195	286	80	429
FDRP	THR	446	48	342	104	485	27	368	109	296	222
FDRP	no THR	97	321	104	337	108	358	200	283	136	374
qFDRP	THR	445	49	336	110	485	27	365	112	274	244
qFDRP	no THR	55	363	62	379	74	392	169	314	83	427
PDR	THR	71	129	68	120	105	124	133	32	100	90
PDR	no THR	62	114	50	133	61	116	79	96	64	141
MHL	THR	28	450	20	409	32	465	63	393	32	464
MHL	no THR	25	382	26	392	25	423	54	399	29	458
Epipoly	THR	26	5	22	6	68	5	67	16	17	75
Epipoly	no THR	8	31	4	30	17	40	23	89	25	65
Entropy	THR	26	5	22	6	69	4	65	18	17	75
Entropy	no THR	8	31	4	30	17	40	23	89	25	65

True State: THR / no THR; Predicted State: THR / no THR. THR–THR: TP; THR–no THR: FN; no THR–THR: FP; no THR–no THR: TN.

Count: 0, 300, 600

Score	True State	**F:** Cell-type heterogeneity		**G:** Cellular contamination		**H:** Allele-specific methylation		**I:** DNA methylation erosion		**J:** Methylation switching domains	
		THR	no THR	THR	no THR	THR	no THR	THR	no THR	THR	no THR
DNAm	THR	527	13	447	127	497	35	451	72	485	29
DNAm	no THR	51	495	73	450	60	436	180	281	77	461
FDRP	THR	503	32	452	121	492	36	411	110	373	137
FDRP	no THR	114	429	125	399	122	375	161	298	114	423
qFDRP	THR	505	30	446	127	493	35	411	110	370	140
qFDRP	no THR	60	483	76	448	64	433	134	325	73	464
PDR	THR	197	203	173	248	233	177	326	40	235	134
PDR	no THR	163	260	118	267	144	234	153	154	154	209
MHL	THR	19	513	22	537	34	489	47	459	28	475
MHL	no THR	20	517	30	479	30	463	49	389	21	499
Epipoly	THR	127	22	106	39	170	7	172	40	45	140
Epipoly	no THR	39	127	31	101	47	143	39	165	44	155
Entropy	THR	128	21	105	40	170	7	172	40	46	139
Entropy	no THR	39	127	31	101	47	143	39	165	46	153

Table S4: Data matrices computed on the healthy blood dataset (blood cohort), the *in-silico* mixed WGBS samples (DEEP hybrid samples), and the cancer example (Ewing sarcoma) with number of rows (sites/regions) and percentage of missing values (NAs) for all considered WSH scores.

WSH Score	blood cohort (RRBS)		DEEP (WGBS)		Ewing sarcoma (RRBS)	
	# sites	% NAs	# sites	% NAs	# sites	% NAs
**FDRP**	1,176,471	2.11%	24,198,968	38.81%	1,227,943	5.75%
**qFDRP**	1,176,471	1.7%	24,198,968	38.81%	1,227,943	5.75%
**PDR**	1,176,471	62.31%	24,198,968	76.66%	1,227,943	65.82%
**MHL**	388,848	19.88%	4,590,846	0%	333,542	27.28%
**Epipolymorphism**	549,129	73.1%	740,216	0%	697,022	83.1%
**Entropy**	549,129	73.41%	740,216	0%	697,022	83.1%

Table S5: Summary of WSH score results on the Ewing sarcoma dataset. The column *Tumor purity prediction* explains cross-validation errors on the sites selected using the LASSO to predict annotated tumor purity levels. CL=cell line, MSC=mesenchymal stem cell, TSS=transcriptional start site, TFBS=transcription factor binding site, MADiff=mean absolute difference

Score	WSH sample groups	Tumor purity prediction	Genome-wide distribution	Differential analysis (MSC vs. Ewing tissue)
**qFDRP**	MSC $>$ Ewing tissue $>$ Ewing CL $>$ Healthy blood	Correlation: 0.966 MADiff: 0.027	lowest in TSS highest in distal enhancers	MSC-hyperheterogeneous sites enriched for Ewing sarcoma specific DNAse-hypersensitive sites
**FDRP**	MSC $>$ Ewing tissue $>$ Ewing CL $>$ Healthy blood	Correlation: 0.972 MADiff: 0.026	lowest in TSS highest in DNAse-hypersensitive sites	MSC-hyperheterogeneous sites enriched for Ewing sarcoma specific DNAse-hypersensitive sites
**PDR**	MSC $>$ Ewing CL $>$ Ewing tissue $>$ Healthy blood	Correlation: 0.894 MADiff: 0.053	lowest in TSS highest in distal enhancers	MSC-hyperheterogeneous sites enriched for different TFBSs (e.g. GATA2, GATA3, STAT3)
**MHL**	MSC $=$ Ewing tissue $=$ Ewing CL $=$ Healthy blood	Correlation: NA MADiff: NA	lowest in TSS highest in CTCF binding sites	MSC-hyperheterogeneous sites enriched for various DNAse-hypersensitive sites
**Epipolymorphism**	MSC $>$ Ewing CL $>$ Ewing tissue $=$ Healthy blood	Correlation: 0.843 MADiff: 0.065	lowest in TSS highest in distal enhancers	MSC-hyperheterogeneous sites enriched for different TFBSs (BRCA1, HNF4) and DNAse-hypersensitive sites
**Entropy**	MSC $>$ Ewing CL $>$ Ewing tissue $=$ Healthy blood	Correlation: 0.910 MADiff: 0.05	lowest in TSS highest in distal enhancers	MSC-hyperheterogeneous sites enriched for different TFBSs (SP1, CHD2) and DNAse-hypersensitive sites
**Methylation**	Healthy blood $>$ Ewing CL $=$ Ewing tissue $>$ MSC	Correlation: 0.920 MADiff: 0.05	lowest in TSS highest in distal enhancers	MSC-hypomethylated sites enriched for various DNAse-hypersensitive sites MSC-hypermethylated sites enriched for different TFBSs (c-MYC, c-Fos) and DNAse-hypersensitive sites linked to Ewing sarcoma

Table S6: Sites selected to predict tumor purity using qFDRP and their genomic annotation (N=26). CGI = CpG island, TFBS = transcription factor binding site, inter = in-between CGIs, shore = CGI shores

Chromosome	Start	End	Strand	Gene name	CGI relation	Ensembl regulatory build
chr1	12648479	12648480	+	AADACL4	inter	
chr1	38196708	38196709	+		inter	
chr1	38813535	38813536	+		inter	
chr1	116376186	116376187	+	ATP1A1	shore	TSS
chr1	165594302	165594303	+		shelf	
chr2	45011168	45011169	+		shore	TSS
chr2	71560568	71560569	+	DYSF	island	
chr2	73324419	73324420	+		inter	
chr2	233084540	233084541	+	INPP5D	inter	TSS
chr3	11290977	11290978	+	ATG7	inter	TSS
chr3	127860038	127860039	+		inter	
chr4	56682124	56682125	+		inter	TSS
chr4	173507461	173507462	+		shore	
chr5	14093992	14093993	+		inter	
chr5	42991519	42991520	+		shore	proximal enhancer
chr5	54632834	54632835	+		inter	
chr6	67094799	67094800	+		inter	proximal enhancer
chr10	3107536	3107537	+	PFKP	shore	proximal enhancer
chr12	24636683	24636684	+		inter	
chr12	34347272	34347273	+		shore	TFBS
chr13	82116448	82116449	+		inter	
chr14	99189256	99189257	+		inter	proximal enhancer
chr14	99236174	99236175	+		inter	proximal enhancer
chr14	103654807	103654808	+		inter	
chr15	67171786	67171787	+	SMAD3	inter	distal enhancer
chr15	95196264	95196265	+		inter	

Table S7: Accuracies obtained from 10-fold cross validation using the sites that consistently had non-zero coefficients in five or more folds in LASSO regression. The average accuracies over 10 different initialization of 10-fold cross validation and the standard deviations over the initializations, as well as the number of selected sites, are shown. MADiff = Mean absolute difference, SD = standard deviation

		FDRP	qFDRP	PDR	MHL	Epipoly	Entropy	Methylation
**Correlation**	**Mean**	0.972	0.966	0.894	NA	0.843	0.910	0.920
	**SD**	0.016	0.012	0.014	NA	0.036	0.026	0.012
**MADiff**	**Mean**	0.026	0.027	0.053	NA	0.065	0.05	0.05
	**SD**	0.001	0.001	0.002	NA	0.001	0.001	0.002
**Number of selected sites**		24	26	19	0	20	16	13

Supplementary Text

Methods

Simulation Setup

General setup We simulated bisulfite sequencing datasets using Sherman (version 0.1.7, [1]) and the human reference genome 'hg38'. Sherman samples reads of a specified length from the reference genome and performs *in-silico* bisulfite conversion. We focused on shorter segments of the genome to create multiple datasets representing different heterogeneity scenarios and technical setups. $1,000$ regions of size 50 kb were randomly selected from the reference genome, discarding chromosomes 22, X and Y due to short length and low CpG content. These regions were selected for each of the simulation scenarios separately. Sherman accepts multiple parameters specifying the final read library. Most notably, `--CG_conversion` is an implicit heterogeneity parameter, which describes the DNA methylation probability for all CpGs. For the simulation scenarios described below, we used 50 bp and 100 bp read length, sampled $25,000/12,500$ reads from the reference genome and included an error rate of 1% at a quality value of 40, unless explicitly stated otherwise (Table S2). Notably, Sherman was not designed for simulating RRBS data in which the start positions of the reads are not uniformly distributed due to the restriction enzyme digestion. We thus implemented an RRBS mode for Sherman in which reads are more likely to use the same start position as any of the reads that were already sampled.

Heterogeneity scenarios We decided to simulate five scenarios: cell-type heterogeneity, cellular contamination, allele-specific methylation (ASM), DNA methylation erosion and DNA methylation switching domains (Figure S1). For each of the scenarios, we sampled $1,000$ regions separately and introduced truly heterogeneous regions (THRs) at random. Each region inherently comprises multiple read subpopulations representing different cell types or alleles according to the simulation scenario. For each of the subpopulations, we introduced a baseline methylation level as either fully methylated or unmethylated (with error level 1%). THRs were then introduced randomly into each of the regions to create positive and negative cases (Figure S2). In case we introduced a THR, the DNA methylation state changed for each of the subpopulations at a random position, thus creating different cell types with varying methylation states. Finally, we merged the subpopulations to create the final region and define the THR as the maximum segment in which any of the subpopulations changed the methylation state. We then benchmarked the WSH scores according to their ability to detect THRs. We used a t-test to compare scores at each CpG position at baseline (i.e. the borders of the subpopulations) with those sites in the THR. A true positive (TP) is determined by testing if the p-value is lower than the given threshold (Figure S2). In contrast, a false negative (FN) would have a p-value higher than the threshold. Since THRs are included at random, about half of the regions will not contain THRs (referred to as negative examples), and we can thus also compute FPs and TNs. Accordingly, ROC curves are computed and we selected a p-value cutoff of 0.01 to compute the confusion matrices in Table S3.
To model cell-type heterogeneity, we first selected the number of simulated cell types between 2 and 10 for each region individually. For each of the cell types, we modeled a separate cell type with a baseline DNA methylation level of either 1 or 0. Each of the cell types (subpopulations) contains a randomly selected subregion, in which the DNA methylation level is set to the inverse with respect to the baseline (Figure S1). After simulating those cell types individually, we merge them to create an artificial cellular mixture. The THR of the cellular mixture is then defined as the union of any of the subregions in the cell types (Figure S2). Furthermore, we computed the average WSH score per region and computed Spearman's rank correlation between the average score and the randomly selected number of cell types over all of the 1,000 regions.
Cellular contamination is modeled by two cell types, one of them being defined as the population (target) cell type and the other as the contaminating cell type. The proportion of the contaminating cell type is randomly selected between 0 and 50%. While the population cell type follows the baseline DNA methylation level, the contaminating cell type contains a subregion with the complementary DNA methylation level. Allele-specific methylation (ASM) can be considered a special case of cellular contamination, in which the proportion of the contaminating cell type is fixed at 50%. We computed Spearman's rank correlation between the average WSH score for all sites per region and the sample purity level.
To model DNA methylation erosion, the stochastic loss of DNA methylation, we first generated fully methylated flanking regions around the THR. Within the THR, we then de-methylated CpGs with probability $1 - \alpha$. This would yield a completely random DNA methylation profile within the THR, which is not realistic. In reality, a subset of cells will stochastically lose DNA methylation, while another subset will remain relatively stable. To model this heterogeneity in selecting a subset of cells for sequencing, we replicated each of the created eroding reads γ-times (Figure S1). We then correlated the simulation parameters to the average WSH score per region.
DNA methylation switching domains (MSDs) were simulated to account for DNA methylation changes that are not due to WSH. Thus, we simulated a single cell type with a baseline DNA methylation level. In the methylation switching domain, the DNA methylation state changes to the complementary level. In this case, negative examples are defined as regions not exhibiting a methylation switch, but constant baseline DNA methylation level.

Technical biases Sherman allows to specify technical parameters of the produced reads. We decided to focus on differences in sequencing coverage, read length, sequencing error level and CpG density. For each of the settings, we sampled $1,000$ regions from the human reference genome and each of these regions is modeled with differences in technical setup.

[1] https://www.bioinformatics.babraham.ac.uk/projects/sherman/

We decided to fix the background heterogeneity parameter to focus only on the technical biases. Regions were either methylated (`--CG_conversion`=5) or unmethylated (`--CG_conversion`=95), according to the average DNA methylation level (62.5%) computed in the blood cohort. Since the remaining 5% of the CpGs show a different DNA methylation level than the background, this leads to a medium overall heterogeneity level. To model sequencing datasets at different read depths, we sampled between 5, 000 and 50, 000 reads of size 50 bp, or between 2, 500 and 25, 000 for 100 bp reads, for each of the regions. For the remaining scenarios, the number of reads was fixed to 25, 000/12, 500. We then counted how often a particular CpG was covered by a sequencing read and correlated this to the WSH of this CpG (site-wise coverage). Similarly, we investigated dependency on CpG density by computing average WSH scores in 50 bp sliding windows and comparing them to the number of CpGs in this window. We changed the read length parameter for the datasets from 40 to 150 bp, keeping the CpG-wise coverage constant. Last, sequencing errors were included into the reads using Sherman's `--error_rate` parameter.

Experimental data

Predicting tumor purity If tumor purity levels are not available either from histopathological or from genetic data, estimating these levels from DNA methylation data can be crucial to account for in downstream analysis. Since WSH scores quantify heterogeneity, they could also be used to estimate tumor purity. Thus, we selected the 81 samples for which we had tumor purity levels estimated from genetic data based on loss of heterozygosity, copy number change and the mutated allele fraction (1) with the method described in (2). Elastic net regression as implemented in the `glmnet` R-package (3; 4) was used to account for the high dimensionality of the problem. We used 10 different initializations of 10-fold nested cross validation to select α and λ simultaneously. α did not have a strong influence on overall model performance and we thus selected $\alpha = 1$ (LASSO), since it returns the least complex models. We then selected those sites that consistently had non-zero coefficients in five or more folds when we executed 10 different initialization of 10-fold cross validation of the LASSO. To validate that we did not find an association by chance, we permuted the sample labels and found that the results were substantially worse. Then, we used the selected sites per WSH score and conducted another 10-fold cross validation using an unregularized linear model with these selected sites to estimate overall performance of the proposed method. We used the selected sites to construct WSH score heatmaps and report them for qFDRP in Table S6, while the cross validated results for all WSH scores are shown in Table S7.

References

[1] Sheffield, N.C., Pierron, G., Klughammer, J., Datlinger, P., Schönegger, A., Schuster, M., Hadler, J., Surdez, D., Guillemot, D., Lapouble, E. et al.. (2017) DNA methylation heterogeneity defines a disease spectrum in Ewing sarcoma. *Nat. Med.*, **23**, 386–395. doi:10.1038/nm.4273.

[2] Chen, X., Stewart, E., Shelat, A.A., Qu, C., Bahrami, A., Hatley, M., Wu, G., Bradley, C., McEvoy, J., Pappo, A. et al.. (2013) Targeting Oxidative Stress in Embryonal Rhabdomyosarcoma. *Cancer Cell*. doi:10.1016/j.ccr.2013.11.002.

[3] Zou, H. and Hastie, T.. (2005) Regularization and Variable Selection via the Elastic Net. *Journal of the Royal Statistical Society*, **67**, 301–320. doi:10.1111/j.1467-9868.2005.00503.x.

[4] Friedman, J., Hastie, T. and Tibshirani, R.. (2010) Regularization Paths for Generalized Linear Models via Coordinate Descent. *Journal of statistical software*, **33**, 1–22.
